# Tunable Porosities and Shapes of Fullerene-Like Spheres

**DOI:** 10.1002/chem.201500692

**Published:** 2015-03-11

**Authors:** Fabian Dielmann, Matthias Fleischmann, Claudia Heindl, Eugenia V Peresypkina, Alexander V Virovets, Ruth M Gschwind, Manfred Scheer

**Affiliations:** [a] Institut für Anorganische Chemie, Universität Regensburg, Institut für Organische Chemie, Universität Regensburg93040 Regensburg (Germany); [b] Nikolaev Institute of Inorganic Chemistry, Siberian Division of RAS, Novosibirsk State UniversityAcad. Lavrentyev str. 3, 630090 Novosibirsk (Russia), Pirogova str. 2, 630090 Novosibirsk (Russia)

**Keywords:** fullerene chemistry, host–guest chemistry, molecular switch, phosphorus, supramolecular chemistry

## Abstract

The formation of reversible switchable nanostructures monitored by solution and solid-state methods is still a challenge in supramolecular chemistry. By a comprehensive solid state and solution study we demonstrate the potential of the fivefold symmetrical building block of pentaphosphaferrocene in combination with Cu^I^ halides to switch between spheres of different porosity and shape. With increasing amount of CuX, the structures of the formed supramolecules change from incomplete to complete spherically shaped fullerene-like assemblies possessing an *I*_h_-C_80_ topology at one side and to a tetrahedral-structured aggregate at the other. In the solid state, the formed nano-sized aggregates reach an outer diameter of 3.14 and 3.56 nm, respectively. This feature is used to reversibly encapsulate and release guest molecules in solution.

## Introduction

Fullerenes, the soluble allotropes of carbon, are robust and rigid spherical molecules both in solution and in the solid state. Only by chemical reactions can they be altered on the surface[1] or opened and closed.[2] However, they are not able to switch between different structures initiated by weak forces. Yet, fullerenes are in the size domain of the nanoscale molecular container molecules. These compounds can be designed to have defined sizes and inner cavities, which make them attractive to an active field of chemistry and materials science. A wide array of molecular container compounds has been synthesized by covalent and noncovalent based syntheses.[3] Such hollow molecules provide confined nano-spaces and serve as molecular containers that can regulate the reactivity and stability of accommodated molecules.[3b–d,f–s] For the bottom-up construction of discrete, well-defined, nanoscale structures the self-assembly approach based on the reversible aggregation of smaller building blocks is feasible. Monitoring of these processes is mostly done by NMR spectroscopy in solution[4] and some stable products have been isolated and structurally characterized. To the best of our knowledge, a comprehensive study of these formation and encapsulation processes in solution and in the solid state is so far absent. Moreover, a rational manipulation of the porosity of big assemblies is exceptional in supramolecular chemistry. Thus, the challenge is to combine the reversibility and the dynamics of encapsulation processes of the container molecules with the shape and rigidity of fullerene-like spheres, a feature that also has not been approached by giant polyoxometalate macromolecules to this extent.[5]

Recently, our group has shown that the combination of copper(I) halides with the *cyclo*-P_5_ complex [Cp*Fe(η^5^-P_5_)] (Cp*=η^5^-C_5_Me_5_) or the *cyclo*-P_4_ complex [Cp′′Ta(CO)_2_(η^4^-P_4_)] (Cp′′=η^5^-C_5_H_3_*t*Bu_2_) provide a powerful tool for the synthesis of carbon-free scaffold structures with nano-sized dimensions.[6] In particular, the fivefold symmetry of the pentaphosphaferrocene imparts a unique approach to spherical molecules with fullerene-like topology. These supramolecules can encapsulate guest molecules like the building block [Cp*Fe(η^5^-P_5_)] itself,[6i,j] *o*-carborane,[6f] C_60_,[6g] or ferrocene.[6c] Based on the template effects, the corresponding 90- or 80-vertex balls are formed around these templates. However, all of the resulting host–guest compounds based on [Cp*Fe(η^5^-P_5_)] have low solubility and therefore no information about the formation pathway can be obtained. In addition, only by applying special reaction conditions can the favored formation of 1D or 2D coordination polymers be avoided.[6j, 7] Thus, the questions arise whether a template-free formation of fullerene-like nanoballs can be achieved, and how can insight into the formation processes and dynamics of the balls in solution be obtained?

**Figure 1:**
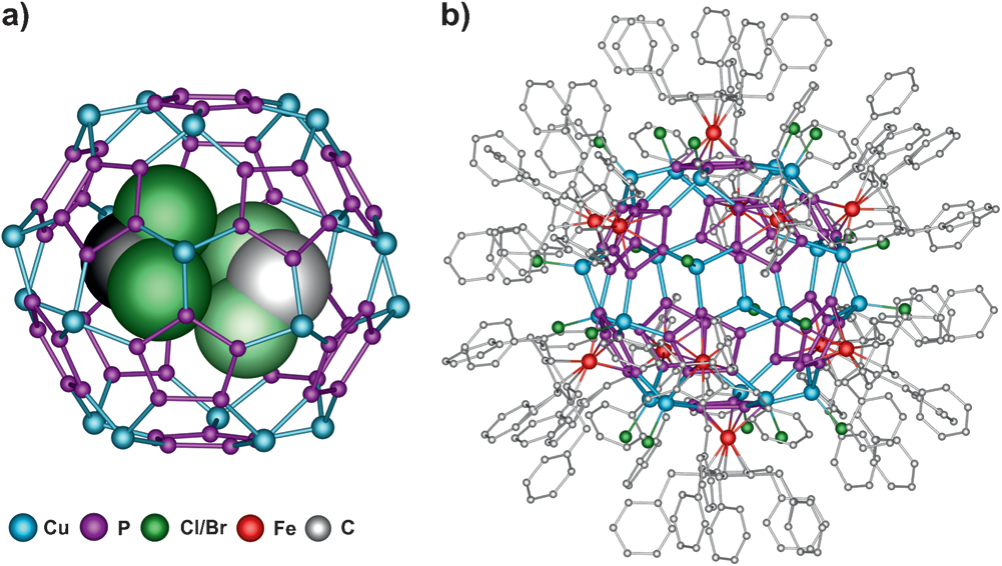
a) Scaffold of the fullerene-like supramolecules 2-Cl and 2-Br with one incorporated molecule CH_2_Cl_2_. A second position of the disordered molecule is shown in lighter shade b) Complete sphere of 2-Cl/2-Br (without H atoms).

**Figure 2:**
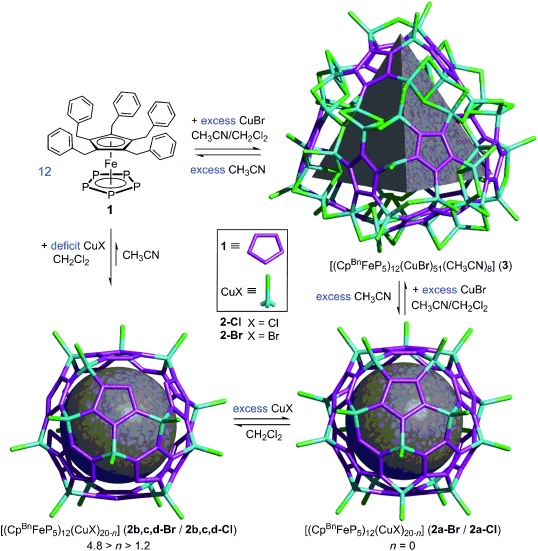
Scheme for the synthesis of 2-Cl/2-Br and 3 and their transformations. The X-ray-derived core scaffolds of the molecular structures of the different supramolecules are depicted.

Herein we report on the reaction of the pentabenzyl derivative [Cp^Bn^Fe(η^5^-P_5_)] (**1**) (Cp^Bn^=η^5^-C_5_(CH_2_C_6_H_5_)_5_)[8] with CuX (X=Cl, Br),[9] for which DOSY experiments show the immediate formation of 80-vertex ball-like molecules in solution. Since **1** is too large to be encapsulated in an 80-vertex spherical system an unprecedented template-free ball formation is achieved (Figure 1 and vide infra). Moreover, due to the high solubility of the products, diffusion experiments for their synthesis with uncontrollable stoichiometry on the bounding surface can be avoided. Therefore, for the first time, the contents of CuX molecules can be controlled by dissolving the appropriate amount of CuX into a solution of **1** needed for a straightforward formation of either incomplete (that means porous) spheres in the desired stoichiometry or complete nano-sized balls, which undergo unprecedented reversible shape rearrangement to a tetrahedral-shaped supramolecule (Figure 2). These switchable molecules are able to encapsulate and release guest molecules. In addition, because the Cp^Bn^ ligand is beneficial for the stability of the supramolecules as well as responsible for their solubility, detailed NMR investigations that monitor the formation process as well as the rearrangement equilibrium have been possible for the first time.

## Results and Discussion

Stirring a solution of **1** in CH_2_Cl_2_ over solid CuX (X=Cl, Br) results in the simultaneous formation of different 80-vertex-type molecules with varying content of CuX. Using different crystallization procedures, the isolation of clusters of the formulae [{Cp^Bn^Fe(η^5^-P_5_)}_12_{CuX}_20−*n*_] (**2-Br**: X=Br, **2-Cl**: X=Cl) was achieved in large quantities (99 % for **2-Br**; 87 % for **2-Cl**). The index *n* (4.8>*n*>0) refers to the incompleteness of the inorganic scaffold, and therefore, isolated sub-compositions can be defined for X=Br: *n*=0: [{Cp^Bn^Fe(η^5^-P_5_)}_12_{CuBr}_20_] (**2 a-Br**); *n*=1.5: [{Cp^Bn^Fe(η^5^-P_5_)}_12_{CuBr}_18.5_] (**2 b-Br**); *n*=1.6: [{Cp^Bn^Fe(η^5^-P_5_)}_12_{CuBr}_18.4_] (**2 c-Br**); *n*=4.6: [{Cp^Bn^Fe(η^5^-P_5_)}_12_{CuBr}_15.4_] (**2 d-Br**); for X=Cl: *n*=0: [{Cp^Bn^Fe(η^5^-P_5_)}_12_{CuCl}_20_] (**2 a-Cl**); *n*=1.2: [{Cp^Bn^Fe(η^5^-P_5_)}_12_{CuCl}_18.8_] (**2 b-Cl**); *n*=2.4: [{Cp^Bn^Fe(η^5^-P_5_)}_12_{CuCl}_17.6_] (**2 c′-Cl**); *n*=3.0: [{Cp^Bn^Fe(η^5^-P_5_)}_12_{CuCl}_17_] (**2 c-Cl**); *n*=4.8: [{Cp^Bn^Fe(η^5^-P_5_)}_12_{CuCl}_15.2_] (**2 d-Cl**). Interestingly, to achieve the complete 80-vertex balls, **2 a-Cl** and **2 a-Br**, small amounts of CH_3_CN are needed (vide infra). In addition, by using an excess of CuBr in CH_3_CN, the supramolecule [{Cp^Bn^Fe(η^5^-P_5_)}_12_{CuBr}_51_{CH_3_CN}_8_] (**3**) was isolated revealing a pseudo-tetrahedral scaffold unprecedented for such pentaphosphaferrocene-based complexes. Because the influence of the halide is rather minor, only the results with CuBr are described herein (for X=Cl see the Supporting Information).

**Figure 3:**
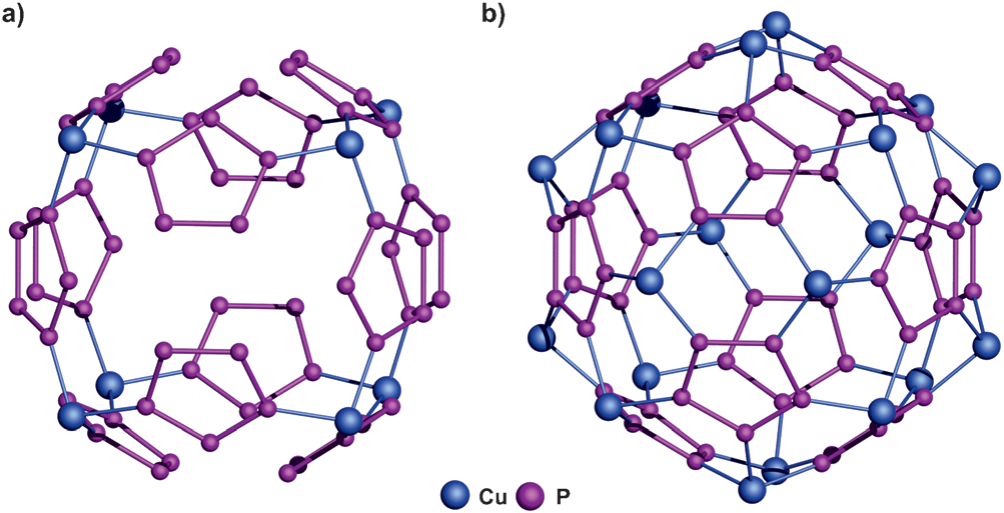
a) Minimal cube-like scaffold of 2-Br involving only the Cu(1) position. b) The complete fullerene-like scaffold of 2 a-Br with fully occupied Cu(1) and Cu(2) positions.

Compound **2 a-Br** crystallizes as black rhombododecahedra in the cubic space group *Pm*
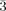
*n*. The whole inorganic scaffold consists of twelve moieties of **1**, which bind to 20 Cu^I^ halide units in a 1,2,3,4,5-coordination mode. The resulting framework contains 80 inorganic core atoms and features a structural motif of twelve five-membered P_5_ and 30 six-membered Cu_2_P_4_ rings (Figure 3 b). Hence, from its core, **2 a-Br** displays an entirely carbon-free analogue of the icosahedral C_80_ molecule. In contrast to the earlier template-controlled approach,[6i,j] the large building block **1** cannot serve as a guest to be encapsulated. Therefore, the inner cavity of **2 a-Br** is now almost empty and only occupied by one solvent molecule CH_2_Cl_2_, which is disordered over 32 positions (Figure 1 a). The void has an almost spherical shape with an inner diameter of about 0.82 nm. Because all of the Cp^Bn^ ligands are pointing away from the fullerene-like scaffold, the outside diameter of this sphere is about 3.14 nm (Figure 1 b), which is approximately 1.0 nm larger than that of the Cp*-derived supramolecule (2.17 nm).[6c]

For the directed crystallization of the complete 80-vertex supramolecule **2 a-Br** CH_2_Cl_2_/CH_3_CN solvent mixtures have to be used, whereas without acetonitrile, the crystallization of incomplete spheres is favored. Compounds **2 b-Br**, **2 c-Br**, and **2 d-Br** crystallize in the same structural type and have systematically vacant inorganic scaffolds. One of the two crystallographically independent CuBr fragments is only partly occupied, which results in similar structures, but in different compositions. The eightfold Cu(1)Br(1) position is always fully occupied, and, being bound to twelve pentaphosphaferrocene complexes, it confines the hypothetical minimal cube-like scaffold (Figure 3 a). The other 12-fold CuBr position, Cu(2)Br(2), leads to **2 a-Br** in case of full occupation, and to **2 b-Br**, **2 c-Br** and **2 d-Br** in case of partial occupation. These additional CuBr fragments can be treated as further support for the scaffold of **2-Br**, because, in the absence of Cu and Br atoms in all of these twelve symmetrically equivalent positions, the integrity of the supramolecule is retained. This feature is the reason for the high stability of **2-Br** in a wide range of the CuBr content.

The minimum number of CuBr units in the inorganic scaffold of all X-ray structural analyses was 15 and, therefore, the hypothetical cube-like scaffold (number of CuBr units=8) was not observed. Under different crystallization conditions, various [{Cp^Bn^Fe(η^5^-P_5_)}_12_{CuBr}_20−*n*_] crystalline phases in which *n*=0, 1.5, 1.6, 4.6 were obtained. This means that the crystals of **2-Br** can be regarded as a solid solution of CuBr-incomplete and/or -complete supramolecules co-crystallized in a particular ratio, even if *n* is an integer. Since the outer surface of the supramolecules is formed by Cp^Bn^ ligands and the vacancies in the individual supramolecule are effectively shielded, the supramolecules can co-crystallize naturally regardless of the CuBr content. The unit cell parameters correlate with neither the nature of the halide nor with the index *n*. Each incomplete supramolecule **2-Br** also incorporates a similarly disordered CH_2_Cl_2_ molecule like **2 a-Br**.

Furthermore, if a large excess of CuBr is used, a supramolecule with a singularly different scaffold for such pentaphosphaferrocene-based complexes is formed, namely [{Cp^Bn^Fe(η^5^-P_5_)}_12_{CuBr}_51_{CH_3_CN}_8_] (**3**) (Figure 2). This compound containing a tetrahedrally shaped structure can be isolated in excellent crystalline yields of up to 96 %.

The supramolecule **3** is partly disordered in the crystal over the twofold axis of the orthorhombic space group *Fddd* (cf. Supporting Information). This disorder can be treated as an overlap of different isomers. One of them is depicted in Figure 2 top right. As in the case of **2-Br**, compound **3** contains twelve pentaphosphaferrocene molecules that are connected to each other by means of polynuclear Cu_*n*_Br_*m*_ fragments containing μ_2_-, μ_3_-, and μ_4_-Br atoms rather than single CuBr units as in **2-Br**. Such a Cu_*m*_ agglomeration was not yet observed for Br-containing supramolecules and was only found in the iodine-containing cluster E_4_@[{Cp*Fe(η^5^-P_5_)}_10_{CuI}_30_{CH_3_CN}_6_] (E=P, As).[6b] The general shape of **3** resembles a giant tetrahedron with one {Cu(CH_3_CN)_2_} group disordered over each of the four corners. All Cu atoms of the main scaffold are coordinated to the P_5_ rings in a η^1^-mode. In addition, the internal cavity is occupied by two {Cu(CH_3_CN)Br} groups, again disordered over four positions, which leaves no accessible volume for guest molecules. The Cu atoms of these groups are additionally coordinated to the *cyclo*-P_5_ rings of **1** in a η^2^-coordination mode, which is unique for this system. A comparable coordination mode was only found in the 1D polymers [Cp*FeP_5_(CuX)_4_(CH_3_CN)_4_⋅0.4 C_6_H_4_Cl_2_]_*n*_ (X=Cl, Br).[7b]

The ideal scaffold of **3** contains 51 Cu and 60 Br positions. If all Br positions are fully occupied, a negative charge and the sum formula [{Cp^Bn^Fe(η^5^-P_5_)}_12_Cu_51_Br_60_{CH_3_CN}_8_]^9−^ would result. However, some terminal and bridging Br positions are statistically vacant, resulting in a neutral [{Cp^Bn^Fe(η^5^-P_5_)}_12_(CuBr)_51_(CH_3_CN)_8_] molecule. The molar ratio of Cu and Br being 1:1 was additionally verified by elemental analysis.

The outside diameter of **3** was determined to be 3.56 nm and is approximately 0.4 nm larger than the diameter of **2-Br**. The topological analysis of the crystal packing made with TOPOS software[10] shows that, despite the dramatic difference in the outer shape, the centers in both **2-Br** and **3** follow an almost ideal body-centered cubic (bcc) motif. In comparison with the face-centered cubic or hexagonal close packing of hard uniform spheres (fcc and hcp), the bcc motif is not the densest one, but the favorable one for soft deformable spheres due to a higher first-sphere coordination number (14 vs. 12).[11]

Due to the fact that the Cp^Bn^ ligands provide good solubility of the supramolecular aggregates, their characterization in solution by high-resolution NMR spectroscopy was possible. Using systematic variations of the experimental conditions, for example, titrations with increasing amounts of CuBr and CD_3_CN or defined starting points by dissolving well-characterized crystals, it was possible to assign the different signals to the supramolecules **2-Br** and **3**. These assignments were mainly based on information from ^1^H and ^31^P chemical shifts as well as line shapes and were supported from data of ^1^H, ^1^H-NOESY/ROESY, ^1^H DOSY, ^1^H,^13^C-HSQC and ^1^H,^13^C-HMBC experiments (for spectra and details see Supporting Information).

The experiments in pure CD_2_Cl_2_ providing the slowest scaffold formation in solution allowed insight into the formation process. Even the averaged diffusion coefficient of all initially detectable species (*D*_**2b–d-Br**_=3.98×10^−10^ m^2^ s^−1^) is close to that of **2 a-Br** (*D*_**2a-Br**_=3.81×10^−10^ m^2^ s^−1^) and far from that of the monomer **1** (*D*_**1**_=10.69×10^−10^ m^2^ s^−1^) indicating a fast formation of the incomplete scaffolds of **2 b–d-Br** on the NMR timescale. The stepwise incorporation of CuBr into this scaffold induced higher intensities of the high-field ^31^P NMR signals of **2 b–d-Br** and a reduction of those at low-field, as expected for a shift from **2 b-Br** to **2 d-Br**. In accordance with the investigations in the solid state, compound **3** was not detected in pure CD_2_Cl_2_, but **2 a CD_2_Cl_2_-Br** was identified in solution. The coexistence of quite large amounts of **2 b–d-Br** beside **2 a CD_2_Cl_2_-Br** even after long reaction times and in the presence of high excess of CuBr indicated a relative high energetic level of **2 a CD_2_Cl_2_-Br** explaining the problems with the crystallization of this species. If traces of CD_3_CN are added, large amounts of **2 a CD_3_CN-Br** are rapidly formed, indicating a much higher stability of **2 a CD_3_CN-Br** compared to **2 a CD_2_Cl_2_-Br**. In the presence of about 1–20 vol % CD_3_CN and two or more equivalents of CuBr, compound **3** was detected in solution. At higher concentrations of CD_3_CN, CuBr units are extracted from the scaffolds and **3** is transformed back to **2 a CD_3_CN-Br** and in the end to **1**.

**Figure 4:**
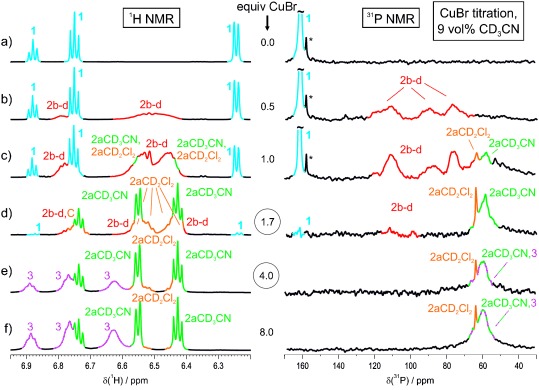
Formation trends and equilibria of 2b–d-Br, 2a CD_2_Cl_2_-Br, 2a CD_3_CN-Br, and 3 in solution. ^1^H and ^31^P spectra of 1 (33 mm) and increasing equivalents of CuBr in CD_2_Cl_2_ with 9 vol % CD_3_CN (^1^H NMR spectra at 298 K and 600 MHz, ^31^P NMR spectra at 300 K and 400 MHz; *=impurity of [Cp^4Bn^Fe(η^5^-P_5_)] (Cp^4Bn^=tetrabenzylcyclopentadienyl)).

The titration row summing up most of these species is shown in Figure 4. CD_2_Cl_2_ with 9 vol % CD_3_CN was used as solvent to allow the detection of **2 a CD_2_Cl_2_-Br**, **2 a CD_3_CN-Br**, and **3** and increasing equivalents of CuBr were added to demonstrate the reversible transformation processes. At 0.5 equivalents CuBr, several very broad signals appeared in the ^1^H and ^31^P NMR spectra indicating the formation of the incomplete supramolecules **2 b–d-Br**. At a CuBr content of 1.0 equivalent, the signals of **2 b–d-Br** increased and small amounts of **2 a CD_2_Cl_2_-Br** and **2 a CD_3_CN-Br** became visible in the ^31^P NMR spectrum. At 1.7 equivalents CuBr, the molar ratio of **2 a-Br**, the signals of **1** and **2 b–d-Br** nearly disappeared, while the signals of **2 a CD_3_CN-Br** and **2 a CD_2_Cl_2_-Br** reached their maximum. Clearly, the large amount of **2 a CD_3_CN-Br** existing beside only tiny amounts of **2 a CD_2_Cl_2_-Br** in 91 % CD_2_Cl_2_ can be seen in Figure 4 d corroborating the higher stability of **2 a CD_3_CN-Br**. The small linewidths of the ^1^H signals of **2 aCD_3_CN-Br** were in accordance with the reduced chemical exchange expected in the highly symmetrical scaffold of **2 a-Br** and the much smaller linewidths of the ^31^P signals of **2 a CD_2_Cl_2_-Br** compared to **2 a CD_3_CN-Br** in combination with the similar ^31^P NMR chemical shift suggested a higher mobility of **Br** units in **2 a CD_3_CN-Br**, most probably caused by CD_3_CN acting a potential ligand (intensity deviations of **2 a CD_2_Cl_2_-Br** in the ^1^H and the ^31^P NMR spectra in Figure 4 e,f are in accordance with the different line broadening due to chemical exchange). Further addition of CuBr led to an increasing formation of **3** in addition to **2 a-Br**.

These formation trends and their complete reversibility were confirmed by dissolving crystals of **2 a-Br** or **3** first in pure CD_2_Cl_2_ and then in CD_2_Cl_2_/CD_3_CN mixtures (for spectra see Supporting Information). In pure CD_2_Cl_2_ providing only a low solubility for CuBr the dissolved crystals of **2 a-Br** led to sharp signals for **2 a CD_2_Cl_2_-Br** as well as small amounts of **2 b–d-Br**. Upon the addition of 3–25 vol % CD_3_CN, **2 a CD_2_Cl_2_-Br** converts to **2 a CD_3_CN-Br** and small signals of **1** appeared confirming the higher stability of **2 a CD_3_CN-Br** than **2 a CD_2_Cl_2_-Br** and the fragmentation at high CD_3_CN concentrations. Crystals of **3** dissolved in pure CD_2_Cl_2_ showed only signals of **2 a CD_2_Cl_2_-Br**, indicating a fragmentation of **3** in the absence of CD_3_CN. Upon addition of 3 vol % of CD_3_CN, strong signals of **3** appeared, which corroborated that CD_3_CN is the key for the stabilization of **3** and conversion to **2 a CD_3_CN-Br**. At higher concentrations of CD_3_CN the availability of CuBr for the supramolecules is reduced leading to reduced signals of **3** and the detection of **2 aCD_3_CN-Br** and **1**. Thus, the presence of exclusively 80-vertex spheres in solution as well as in solid state is ensured by avoiding acetonitrile during the formation and crystallization processes. Even though a solution of **3** is always accompanied by some amounts of **2 a-Br**, the sole and reproducible crystallization of **3** without any traces of **2 a-Br** is provided by using the optimized conditions of 5.5 equivalents of CuBr and 15 vol % CH_3_CN. To conclude, the appropriate solvent mixture and CuBr content is absolutely crucial for the directed formation of the supramolecules. The required conditions are illustrated in Figure 2. Furthermore, these observations illustrate the potential to control the reversible switch between the different supramolecular morphologies.

Since the supramolecules have defined inner cavities and their formation pathway and behavior in solution is dynamic, they become ideal for use as nanometer-sized containers for small molecules. Thus, the incorporation of ferrocene was studied, because this potential guest has the appropriate size and its incorporation was already achieved by using [Cp*Fe(η^5^-P_5_)] as a building block.[5c]

In CD_2_Cl_2_ that contained only **1**, 2 equivalents of CuBr, and 0.25 equivalents of ferrocene, 1–2 vol % CD_3_CN was added to the reaction mixture, which promoted the formation of **2 a CD_3_CN-Br**, **2 a CD_2_Cl_2_-Br** and **3**, similar to the above described experiments. In addition to the singlet of free ferrocene at 4.15 ppm, three small singlets at 0.66, 0.31, and 0.13 ppm were detected in the ^1^H spectrum; these led to diffusion coefficients typical for **2-Br** and were assigned to **FeCp_2_@2 aCD_3_CN**, **FeCp_2_@2 aCD_2_Cl_2_** and **FeCp_2_@2 b–d** (for details and spectra see Supporting Information). Their upfield shifts of 3.49–4.02 ppm compared to free ferrocene were in the same range as observed for the encapsulation of *o*-carborane[6f] and C_60_[6g] in similar pentaphosphaferrocene-derived capsules. The inclusion of ferrocene inside **2 aCD_3_CN-Br** was corroborated by NOESY experiments providing NOE cross peaks between ferrocene in **FeCp_2_@2 aCD_3_CN** and the benzylic methylene protons of **2 aCD_3_CN-Br**, but none to the aromatic protons of **2 aCD_3_CN-Br** at the surface of the supramolecule. Neither high-field-shifted signals correlating with the intensity changes of **3** nor NOESY cross peaks between incorporated ferrocene and **3** were observed, which is consistent with the cavity of **3** not being suitable to act as a host for ferrocene. Furthermore, no exchange peaks between encapsulated ferrocene and free ferrocene were detected in ^1^H, ^1^H NOESY, and ROESY spectra, indicating an exchange rate below the NMR timescale. Finally, the incorporation was proven by X-ray structural analysis of a product isolated in 98 % yield.

The ferrocene molecules were found in the inner cavity of the solid structure of (FeCp_2_)@[(Cp^Bn^FeP_5_)_12_(CuBr)_20−*n*_] with *n*=1.3 (**(FeCp_2_)@2-Br**) (for figures and **(FeCp_2_)@2-Cl**, see Supporting Information). The guest molecule is fixed in the cavity of the container through π–π stacking interactions between the Cp rings of ferrocene and the *cyclo*-P_5_ units of the host molecule similarly to (FeCp_2_)@[{Cp*Fe(η^5^-P_5_)}_12_{CuCl}_20_].[6c] They lead to Cp⋅⋅⋅P_5_ interplane distances of 4.05 Å.

The rapid formation of the different species in the presence of low amounts of CD_3_CN indicated that there is no kinetic hindrance for their transformation, but that the clusters represent different thermodynamic minima, which are separated by a relatively low energetic barrier. Therefore we investigated their potential to act as supramolecular switches. With ferrocene as guest, the potential of in- and exclusion of guests was also elucidated.

**Figure 5:**
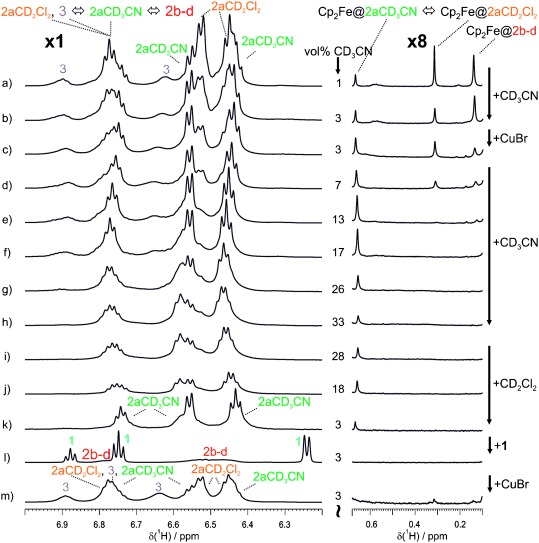
a)–m) Titration ^1^H spectra of the self-assembling supramolecular switch with incorporated guests composed of 1, 2.0 equivalents CuBr, and 0.25 equivalents ferrocene in CD_3_CN/CD_2_Cl_2_ mixtures, each at 298 K and 600 MHz. Starting from mixture a) CD_3_CN, CuBr, CD_2_Cl_2_ or 1 were added, see arrows. Due to this procedure the relative, not the absolute signal intensities, have to be considered (see text).

To switch between the different container molecules (**2 b–d-Br, 2 a CD_2_Cl_2_-Br, 2 a CD_3_CN-Br and 3**), compound **1** and ferrocene were stirred over solid CuBr in CD_2_Cl_2_ and CD_3_CN. Subsequently, CuBr, CD_2_Cl_2_, or **1** were added in a titration row (Figure 5). The relative intensity changes of the encapsulated ferrocene signals **FeCp_2_@2 a CD_3_CN-Br**, **FeCp_2_@2 a CD_2_Cl_2_-Br**, and **FeCp_2_@2 b–d-Br** followed directly the formation trends of their container molecules, while the absolute intensities are affected by the dilution effect (e.g., the percentage of encapsulated guests is similar in Figure 5^5^ j and 5 f). Thus, addition of CuBr leads to a shift from **FeCp_2_@2 b–d-Br** to; **FeCp_2_@2 a CD_2_Cl_2_-Br** (Figure 5 c) and addition of CD_3_CN to an increase of **FeCp_2_@2 a CD_3_CN-Br** at the expense of; **FeCp_2_@2 a CD_2_Cl_2_-Br** and **3** (Figure 5 d,e). At CD_3_CN contents higher than 17 vol % (Figure 5 g), a reduction of the relative signal intensity of **FeCp_2_@2 a CD_3_CN-Br** was observed indicating a partial release of some of the encapsulated ferrocenes from their hosts **2 a CD_3_CN-Br** due to the accelerated mobility of CuBr, which is in agreement with the observed partial rearrangement of the supramolecules at high CD_3_CN contents. After a reduction of the CD_3_CN content back to 18 vol %, the relative amount of **Cp_2_Fe@2 a CD_3_CN-Br** increased again (Figure 5 i), indicating the reversibility of these inclusion processes. Also by addition of **1** leading to more rudimental scaffolds ferrocene can be released (Figure 5 l), while by subsequent addition of CuBr ferrocene is again encapsulated (Figure 5 m) corroborating the reversibility of the process (again the absolute intensity change between Figure 5 m and 5 a–c is caused by dilution).

From these experiments, we have demonstrated the potential of the supramolecules to switch among each other and additionally shown the inclusion and exclusion of a ferrocene guest. The switch from **3** to **2-Br** was realized by an increase of the content of acetonitrile. The reversibility of the system and thus the reverse transformation from **2-Br** to **3** was demonstrated by the subsequent reduction of the CD_3_CN content. Furthermore, by the addition of an excess of **1** the included ferrocene was released from **2-Br**. The re-formation of the clusters by the addition of CuBr successfully reconstitutes the supramolecular switch.

## Conclusions

In summary, a combined solution NMR and solid-state X-ray study is presented describing soluble, quantitative self-assembling, and switchable nanometer-sized supramolecules, composed of fivefold-symmetrical building blocks, [Cp^Bn^Fe(η^5^-P_5_)] (**1**), and CuX (X=Cl, Br), with the ability to incorporate guest molecules. For the first time a controlled access is possible to either incomplete (that is, porous) spheres (**2 b–d**), or to complete nano-sized balls (**2 a**). Compounds **2 a** undergo reversible and selective shape rearrangement to an unprecedented tetrahedral-shaped supramolecule **3**, which reaches an outer diameter of 3.56 nm. The template-independent formation of the fullerene-like nanoballs was found to depend on the CuX content and the overall concentration of the CD_3_CN/CD_2_Cl_2_ mixture. The manipulation of the solvent mixture delivers a reversible self-assembling supramolecular switch, which allows for the selective incorporation of guests, as exemplified by ferrocene. In solution this guest can be easily removed and again incorporated from the host by addition of an excess of **1** or CuBr, respectively, for several cycles. These features go much beyond the known properties of fullerenes and show therefore the great potential of this non-carbon-based system in supramolecular chemistry.

## Experimental Section

All reactions were performed under an inert atmosphere of dry nitrogen or argon with standard vacuum, Schlenk, and glove-box techniques. Solvents were purified and degassed by standard procedures. Commercially available chemicals were used without further purification. Complex **1** was prepared as described before.[8] ESI-MS spectra were measured on a ThermoQuest Finnigan MAT TSQ 7000 mass spectrometer, and EI-MS spectra on a Finnigan MAT SSQ 710A mass spectrometer. The C, H, N analyses were determined on a Vario EL III apparatus, the elemental analysis of all elements of **3** was done by the Institute of Inorganic Chemistry, Technische Universität München.

NMR spectra were recorded on a Bruker Avance 400 MHz spectrometer equipped with a BBO probe with z-gradient and BVT 2000 temperature control unit at 300 K, a Bruker Avance III 600 MHz spectrometer equipped with a TBI ^1^H/^31^P-BB z-gradient probe and BVT 3000 unit at 300 K and an Avance III 600 MHz spectrometer equipped with a 5 mm TCI z-gradient cryo probe and BVT 3000 unit at 298 K. The spectra were processed with the Bruker program Topspin® and the diffusion coefficients were calculated with the Bruker software *T1/T2* package. All experimental diffusion coefficients were within a standard deviation of ±3 % and are stated as temperature- and viscosity-corrected diffusion coefficients in the manuscript. For the calibration of the ^31^P chemical shifts, the *Ξ* value corresponding to TMS was applied. For the calibration of the ^1^H and ^13^C chemical shifts and for the temperature- and viscosity-correction of the diffusion coefficients, TMS (tetramethylsilane) was added to each sample. All ^1^H-diffusion measurements were performed with the convection suppressing DSTE (double stimulated echo) pulse sequence, developed by Müller and Jerschow[12] in a pseudo 2D mode. For each experiment, 2 dummy scans and 16 scans were used with a relaxation delay of 2 s. Sinusoidal shapes were used for the gradients and a linear gradient ramp with either 12 or 16 increments between 5 and 95 % of the maximum gradient strength was applied for the diffusion relevant gradients. For the homospoil gradients, 7.046, 10.700, and 9.165 G cm^−1^ were applied for HS_1_, HS_2_, and HS_3_. The length of the gradient pulse *δ* was adjusted for every species in the sample to achieve appropriate signal attenuation curves. As a result, a *δ* of 1.5 ms for TMS, 2.2 ms for the monomer and 3.2 ms for the supramolecules was used in most cases. A diffusion time Δ of 50 ms was used for all experiments. Sample concentrations of 33 mm of **1** (15 mg in 0.6 mL solvent) in CD_2_Cl_2_ or CD_2_Cl_2_/CD_3_CN mixtures were typically applied for the NMR measurements. The different samples were prepared by stirring of **1** together with CuX (X=Cl, Br) in the respective solvent or solvent mixture. After a reaction time of 1 h each sample was filtered and then characterized. Assignments of proton, carbon, and phosphorous resonances of the species were obtained by one- and two-dimensional NMR spectra (^1^H, ^31^P, ^1^H,^13^C-HSQC, ^1^H,^13^C-HMBC, ^1^H,^1^H-ROESY and ^1^H,^1^H-NOESY (mixing times of 600, 350 and 100 ms) spectra.

**Synthesis of 2 a-Br**: Complex **1** (100 mg, 0.14 mmol) was dissolved in toluene (5 mL) in a long, thin, Schlenk tube. A solvent mixture of toluene/MeCN (3:1, 1.5 mL) and subsequently a solution of CuBr (40 mg, 0.28 mmol) in MeCN (2.5 mL) were layered on top of the resulting green solution. The reaction mixture was allowed to stand in an undisturbed area at room temperature in the dark. Within one month dark crystals of **2 a-Br** were formed. The mother liquor was decanted and the crystals were washed with toluene (2×4 mL) and Et_2_O (3×5 mL) and dried under vacuum at room temperature to afford 132 mg (99 %) of **2 a-Br**. Instead of toluene, CH_2_Cl_2_, 1,2-dichorobenzene or 1,2-difluorobenzene can also be used as solvent. However, crystals of **2 a** are not formed before layering the mixture with a less polar solvent like Et_2_O or hexane. ^1^H NMR (CD_2_Cl_2_, 400.13 MHz, 300 K): *δ*=6.75 (m, 60 H; *H*^*para*^), 6.56 (m, 120 H; *H*^*meta*^), 6.44 (m, 120 H; *H*^*ortho*^), 5.09 ppm (m, 120 H; C*H*_2_); ^31^P{^1^H} NMR (CD_2_Cl_2_, 161.98 MHz, 300 K): *δ*=63.8 ppm (s); positive ion ESI-MS (CH_2_Cl_2_/MeCN): *m*/*z* (%)=2672.1 (2) [(Cp^Bn^FeP_5_)_3_(CuBr)_3_Cu]^+^, 2530.1 (2) [(Cp^Bn^FeP_5_)_3_(CuBr)_2_Cu]^+^, 2386.8 (1) [(Cp^Bn^FeP_5_)_3_(CuBr)Cu]^+^, 1802.6 (5) [(Cp^Bn^FeP_5_)_2_(CuBr)_2_Cu]^+^, 1659.6 (16) [(Cp^Bn^FeP_5_)_2_(CuBr)Cu]^+^, 1515.6 (46) [(Cp^Bn^FeP_5_)_2_Cu]^+^, 1076.6 (6) [(Cp^Bn^FeP_5_)(CuBr)_2_Cu]^+^, 973.9 (7) [(Cp^Bn^FeP_5_)(CuBr)(MeCN)Cu]^+^, 932.9 (10) [(Cp^Bn^FeP_5_)(CuBr)Cu]^+^, 830.1 (100) [(Cp^Bn^FeP_5_)Cu(MeCN)]^+^, 789.0 (60) [(Cp^Bn^FeP_5_)Cu]^+^_;_ negative ion ESI-MS (CH_2_Cl_2_/MeCN): *m*/*z* (%)=366.5 (4) [Cu_2_Br_3_]^−^, 222.8 (100) [CuBr_2_]^−^; IR (KBr): 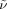
=3105 (vw; CH), 3085 (w; CH), 3060 (m; CH), 3028 (m; CH), 3004 (vw; CH), 2917 (w; CH_2_), 1948 (w), 1884 (w), 1803 (w), 1624 (m), 1603 (s; CC), 1495 (vs; CC), 1454 (s; *δ*(CH_2_)), 1446 (s; *δ*(CH_2_)), 1077 (m), 1030 (m), 732 (vs; *δ*(Ph)), 696 (vs; *δ*(Ph)), 517 (w), 489 (m), 463 cm^−1^(w); elemental analysis calcd (%) for (C_40_H_35_FeP_5_)_12_(CuBr)_20_(CH_2_Cl_2_) (11 586.04 g mol^−1^): C 49.50, H 3.64; found: C 49.41, H 3.58.

**Synthesis of 2 b–d-Br**: A solution of **1** (40 mg, 0.055 mmol) in CH_2_Cl_2_ (8 mL) was stirred over solid CuBr (24 mg, 0.17 mmol) for 4 h (more incomplete spheres, **2 d-Br**) to 3 days (more complete spheres, **2 b-Br**). The resulting deep red solution was transferred into a thin Schlenk tube and layered with toluene (8 mL). Within three weeks large single crystals of **2 b–d** were formed. They were isolated, washed with toluene (2×2 mL) and pentane (2×5 mL) and dried under vacuum first at room temperature and finally for 30 min at 70 °C to afford 38 mg (71 %) of **2 b–d**. Different molar ratios of CuBr also influence the completeness of the supramolecules: A deficit of CuBr (e.g. 5 mg, 0.03 mmol) leads to more incomplete spheres (**2 d-Br**). ^1^H NMR (CD_2_Cl_2_, 600 MHz, 298 K): *δ*=6.80 (m {br}, 60 H; *H*^*para*^), 6.40–6.60 (m {br}, 240 H; *H^meta^, H*^*ortho*^), 5.10 (s {br}, 120 H; C*H*_2_), 4.84 (s {br}, 120 H; C*H*_2_), 4.67 ppm (s {br}, 120 H; C*H*_2_); ^31^P{^1^H} NMR (CD_2_Cl_2_, 400 MHz, 300 K): *δ*=140 (s {br}), 90 (s {br}), 79 ppm (s {br}); elemental analysis calcd (%) for (C_40_H_35_FeP_5_)_12_(CuBr)_18_(CH_2_Cl_2_) (11 384 g mol^−1^): C 50.75, H 3.74; found: C 50.99, H 3.75.

**Synthesis of 3**: In a schlenk tube complex **1** (100 mg, 0.14 mmol) was dissolved in CH_2_Cl_2_ (25 mL) to give an intensive green solution; a colorless solution of CuBr (110 mg, 0.78 mmol) in CH_3_CN (4 mL) was then added. The reaction mixture immediately turns red-brown and was stirred for a further two hours. Next a CH_2_Cl_2_/CH_3_CN (2:1, 2 mL) solvent mixture and subsequently toluene (20 mL) were layered on top of it. The reaction mixture was allowed to stand in an undisturbed area at room temperature. Within two days the crystallization process starts with the formation of small red rods of **3** at the phase boundary. After complete diffusion, the almost completely discolored mother liquor was decanted, and the crystals were washed with hexane (4×5 mL) and dried under vacuum at room temperature to afford **3** (180 mg, 0.011 mmol, 96 %). A higher molar ratio of CuBr (e.g., 6 equiv; 120 mg) with respect to **1** leads to the crystallization of a small amount of CuBr in addition to **3**, a lower molar ratio of CuBr (e.g., 5 equiv; 100 mg) on the other hand leads to the crystallization of **2** in addition to **3**. Therefore, the molar ratio of 5.5 equivalents CuBr (110 mg) proved to be the ideal stoichiometry for the selective crystallization of **3**. ^1^H NMR (CD_2_Cl_2_, 600 MHz, 298 K): *δ*=6.89 (m {br}, 60 H, *H*^*para*^), 6.76 (m {br}, 120 H, *H*^*meta*^), 6.63 (m {br}, 120 H, *H*^*ortho*^), 4.57 ppm (m, {br}, 120 H, C*H*_2_); ^31^P{^1^H} NMR (CD_2_Cl_2_, 400 MHz, 300 K): *δ*=59.8 ppm (s, {br}); elemental analysis calcd (%) for (C_40_H_35_FeP_5_)_12_(CuBr)_51_(CH_3_CN)_3_: (16 156 g mol^−1^): C 36.13, H 2.68, Br 25.2, Cu 20.0, Fe 4.15, N 0.26, P 11.50; found: C 35.94, H 2.80, Br 25.0, Cu 19.5, Fe 4.13, P 10.71.
