# Supplementary material for: Tunable Porosities and Shapes of Fullerene-Like Spheres
Source: Chemistry. 2015 Mar 11;21(16):6208–14. doi: 10.1002/chem.201500692 (PMC4464546; doi:10.1002/chem.201500692)
Supplement: Supplementary file 1 [file chem0021-6208-sd1.pdf]

CHEMISTRY
A European Journal

Supporting Information

Tunable Porosities and Shapes of Fullerene-Like Spheres

Fabian Dielmann,[a] Matthias Fleischmann,[a] Claudia Heindl,[a] Eugenia V. Peresypkina,[b] Alexander V. Virovets,[b] Ruth M. Gschwind,[a] and Manfred Scheer*[a]

chem_201500692_sm_miscellaneous_information.pdf

SUPPORTING INFORMATION

Tunable Porosities and Shape of Fullerene-like Spheres

Fabian Dielmann, Matthias Fleischmann, Claudia Heindl, Eugenia V. Peresypkina, Alexander V. Virovets, Ruth M. Gschwind, Manfred Scheer

1. **Experimental Part** 1
2. **X-ray Structure Analysis** 6
3. **NMR Experiments in Solution** 16

1. Experimental Part

All reactions were performed under an inert atmosphere of dry nitrogen or argon with standard vacuum, Schlenk, and glove-box techniques. Solvents were purified and degassed by standard procedures. Commercially available chemicals were used without further purification. Complex **1** was prepared as described before. ESI-MS spectra were measured on a ThermoQuest Finnigan MAT TSQ 7000 mass spectrometer, EI-MS spectra on a Finnigan MAT SSQ 710A mass spectrometer. The C, H, N analyses were determined on a Vario EL III apparatus, the elemental analysis of all elements of **3** was done by the Institute of Inorganic Chemistry, Technische Universität München.

1.1 Synthesis of [{$Cp^{Bn}Fe(\mu_6\text{-}\eta^5:\eta^1:\eta^1:\eta^1:\eta^1:\eta^1\text{-}P_5)$}$_{12}${CuCl}$_{20}$] (2a-Cl)

Complex **1** (100 mg, 0.138 mmol) was dissolved in toluene (10 mL) in a long thin Schlenk tube. On top of the resulting green solution were layered first 3 mL of a solvent mixture of toluene/MeCN (3:1) and subsequently a solution of CuCl (27 mg, 0.273 mmol) in MeCN (5 mL). The reaction mixture was stored in an undisturbed area at room temperature in the dark. Within three weeks dark crystals of **2a-Cl** were formed. The mother liquor was decanted and layered with Et_2O (18 mL), whereby a second crop of **2a-Cl** was obtained. The crystals were isolated,

washed with toluene (2 × 2 mL) and Et_2O (3 × 5 mL) and dried under vacuum at room temperature to afford 107 mg (87 %) of **2a-Cl**.

Instead of toluene, CH_2Cl_2 can also be used as solvent. Thereby, crystals of **2a-Cl** are not formed before layering the mixture with Et_2O, due to the better solubility of **2a-Cl** in the CH_2Cl_2/MeCN mixture. Compound **2a-Cl** can be isolated in good yield (75 %), but the obtained crystals are of bad quality for a single crystal X-ray structure analysis.

Analytical data of $[\{Cp^{Bn}Fe(\mu_6\text{-}\eta^5\text{:}\eta^1\text{:}\eta^1\text{:}\eta^1\text{:}\eta^1\text{:}\eta^1\text{-}P_5)\}_{12}\{CuCl\}_{20}]$ (**2a-Cl**):

Yield: 107 mg (87 %)

^{1}H NMR (CD_2Cl_2, 400.13 MHz, 300 K): δ [ppm] = 6.76 (m {br}, 60 H; H^{para}), 6.51 (m {br}, 240 H; H^{meta}, H^{ortho}), 4.94 (m {br}, 120 H; CH_2)

^{31}P{^{1}H} NMR (CD_2Cl_2, 161.98 MHz, 300 K): δ [ppm] = 71.5 (s {br})

MAS ^{31}P{^{1}H} NMR (C_6D_6, 161.98 MHz, 300 K): δ [ppm] = 74.0 (s {br}, $\omega_{1/2}$ = 4700 Hz) with broad shoulder at 122.5 (s {br}, $\omega_{1/2}$ = 5900 Hz),

Positive ion ESI-MS (CH_2Cl_2): *m/z* (%) = 1515.6 (20) $[(Cp^{Bn}FeP_5)_2Cu]^+$, 788.9 (100) $[(Cp^{Bn}FeP_5)Cu]^+$

MALDI-TOF (DCTB matrix): *m/z* (%) = 1614.9 (68) $[(Cp^{Bn}FeP_5)_2Cu_2Cl]^+$, 1515.0 (100) $[(Cp^{Bn}FeP_5)_2Cu]^+$

IR (KBr): $\tilde{\nu}$ [cm^{-1}] = 3105 (vw; CH), 3085 (w; CH), 3060 (m; CH), 3028 (m; CH), 3004 (vw; CH), 2918 (w; CH_2), 1949 (w), 1885 (w), 1803 (w), 1624 (m), 1603 (s; CC), 1495 (vs; CC), 1455 (s; $\delta(CH_2)$), 1446 (s; $\delta(CH_2)$), 1076 (m), 1030 (m), 732 (vs; δ(Ph)), 696 (vs; δ(Ph)), 517 (w), 489 (m), 463 (w)

Elemental analysis: Calculated (%) for $(C_{40}H_{35}FeP_5)_{12}(CuCl)_{20}(CH_2Cl_2)$ (10782 g/mol): C 53.58, H 3.94; found: C 53.29, H 3.95

1.2 Synthesis of $[\{Cp^{Bn}Fe(\mu_6\text{-}\eta^5\text{:}\eta^1\text{:}\eta^1\text{:}\eta^1\text{:}\eta^1\text{:}\eta^1\text{-}P_5)\}_{12}\{CuCl\}_{20\text{-}n}]$ (2b,c,d-Cl)

A solution of **1** (40 mg, 0.055 mmol) in CH_2Cl_2 (8 mL) was stirred over solid CuCl (16 mg, 0.16 mmol) for 2 hours (more incomplete spheres, **2d-Cl**) to 3 days (more complete spheres, **2b-Cl**). The resulting deep red solution was transferred into a thin Schlenk tube and layered with toluene (8 mL). Within three weeks large single crystals of **2b,c,d-Cl** were formed. They were

isolated, washed with toluene (2 × 2 mL) and pentane (2 × 5 mL) and dried under vacuum, first at room temperature and finally for 30 minutes at 70 °C to afford 26 mg (53 %) of **2b,c,d-Cl**. Different molar ratios of CuCl also influence the completeness of the supramolecules: A deficit of CuCl (e.g. 3 mg, 0.03 mmol) leads to more incomplete spheres (**2d-Cl**).

Analytical data of [{$Cp^{Bn}Fe(\mu_6\text{-}\eta^5:\eta^1:\eta^1:\eta^1:\eta^1:\eta^1\text{-}P_5)$}$_{12}${CuCl}$_{20}$] (**2b,c,d-Cl**):

Yield: 26 mg (53 %)

^{1}H NMR (CD_2Cl_2, 400.13 MHz, 300 K): δ [ppm] = 6.80 (m {br}, 60 H, $\omega_{1/2}$ = 40 Hz; H^{para}), 6.40-6.60 (m {br}, 240 H, $\omega_{1/2}$ = 70 Hz; H^{meta}, H^{ortho}), 5.0 (s {br}, 120 H, CH_2)

^{31}P{^{1}H} NMR (CD_2Cl_2, 400 MHz, 300 K): δ [ppm] = 110 (s {br})

1.3 Synthesis of [{$Cp^{Bn}Fe(\mu_6\text{-}\eta^5:\eta^1:\eta^1:\eta^1:\eta^1:\eta^1\text{-}P_5)$}$_{12}${CuBr}$_{20}$] (2a-Br)

Complex **1** (100 mg, 0.14 mmol) was dissolved in toluene (5 mL) in a long thin Schlenk tube. On top of the resulting green solution were layered first 1.5 mL of a solvent mixture of toluene/MeCN (3:1) and subsequently a solution of CuBr (40 mg, 0.28 mmol) in MeCN (2.5 mL). The reaction mixture was allowed to stand in an undisturbed area at room temperature in the dark. Within one month dark crystals of **2a-Br** were formed. The mother liquor was decanted and the crystals were washed with toluene (2 × 4 mL) and Et_2O (3 × 5 mL) and dried under vacuum at room temperature to afford 132 mg (99 %) of **2a-Br**.

Instead of toluene, CH_2Cl_2, 1,2-dichorobenzene or 1,2-difluorobenzene can also be used as solvent. Thereby, crystals of **2a** are not formed before layering the mixture with a less polar solvent like Et_2O or hexane.

Analytical data of [{$Cp^{Bn}Fe(\mu_6\text{-}\eta^5:\eta^1:\eta^1:\eta^1:\eta^1:\eta^1\text{-}P_5)$}$_{12}${CuBr}$_{20}$] (**2a-Br**):

Yield: 132 mg (99 %)

^{1}H NMR (CD_2Cl_2, 400.13 MHz, 300 K): δ [ppm] = 6.75 (m, 60 H; H^{para}), 6.56 (m, 120H; H^{meta}), 6.44 (m, 120 H; H^{ortho}), 5.09 (m, 120 H; CH_2)

^{31}P{^{1}H} NMR (CD_2Cl_2, 161.98 MHz, 300 K): δ [ppm] = 63.8 (s)

Positive ion ESI-MS (CH_2Cl_2/MeCN): *m/z* (%) = 2672.1 (2) [$(Cp^{Bn}FeP_5)_3(CuBr)_3Cu]^+$, 2530.1 (2) [$(Cp^{Bn}FeP_5)_3(CuBr)_2Cu]^+$, 2386.8 (1) [$(Cp^{Bn}FeP_5)_3(CuBr)Cu]^+$, 1802.6 (5) [$(Cp^{Bn}FeP_5)_2(CuBr)_2Cu]^+$, 1659.6 (16) [$(Cp^{Bn}FeP_5)_2(CuBr)Cu]^+$, 1515.6 (46) [$(Cp^{Bn}FeP_5)_2Cu]^+$,

1076.6 (6) [($Cp^{Bn}FeP_5$)($CuBr)_2Cu]^+$, 973.9 (7) [($Cp^{Bn}FeP_5$)(CuBr)(MeCN)$Cu]^+$, 932.9 (10) [($Cp^{Bn}FeP_5$)(CuBr)$Cu]^+$, 830.1 (100) [($Cp^{Bn}FeP_5$)$Cu]^+$, 789.0 (60) [($Cp^{Bn}FeP_5$)$Cu]^+$

Negative ion ESI-MS (CH_2Cl_2/MeCN): *m/z* (%) = 366.5 (4) $[Cu_2Br_3]^+$, 222.8 (100) $[CuBr_2]^+$

IR (KBr): $\tilde{\nu}$ [cm^{-1}] = 3105 (vw; CH), 3085 (w; CH), 3060 (m; CH), 3028 (m; CH), 3004 (vw; CH), 2917 (w; CH_2), 1948 (w), 1884 (w), 1803 (w), 1624 (m), 1603 (s; CC), 1495 (vs; CC), 1454 (s; $\delta(CH_2)$), 1446 (s; $\delta(CH_2)$), 1077 (m), 1030 (m), 732 (vs; δ(Ph)), 696 (vs; δ(Ph)), 517 (w), 489 (m), 463 (w)

Elemental analysis: Calculated (%) for $(C_{40}H_{35}FeP_5)_{12}(CuBr)_{20}(CH_2Cl_2)$ (11586.04 g/mol): C 49.50, H 3.64; found: C 49.41, H 3.58

1.4 Synthesis of [{$Cp^{Bn}Fe(\mu_6\text{-}\eta^5:\eta^1:\eta^1:\eta^1:\eta^1:\eta^1\text{-}P_5)$}$_{12}${CuBr}$_{20\text{-}n}$] (2b,c,d-Br)

A solution of **1** (40 mg, 0.055 mmol) in CH_2Cl_2 (8 mL) was stirred over solid CuBr (24 mg, 0.17 mmol) for 4 hours (more incomplete spheres, **2d-Br**) to 3 days (more complete spheres, **2b-Br**). The resulting deep red solution was transferred into a thin Schlenk tube and layered with toluene (8 mL). Within three weeks large single crystals of **2b,c,d** were formed. They were isolated, washed with toluene (2 × 2 mL) and pentane (2 × 5 mL) and dried under vacuum first at room temperature and finally for 30 minutes at 70 °C to afford 38 mg (71 %) of **2b,c,d**.

Different molar ratios of CuBr also influence the completeness of the supramolecules: A deficit of CuBr (e.g. 5 mg, 0.03 mmol) leads to more incomplete spheres (**2d-Br**).

Analytical data of [{$Cp^{Bn}Fe(\mu_6\text{-}\eta^5:\eta^1:\eta^1:\eta^1:\eta^1:\eta^1\text{-}P_5)$}$_{12}${CuBr}$_{20\text{-}n}$] (**2b-d**):

Yield: 38 mg (71 %)

^{1}H NMR (CD_2Cl_2, 600 MHz, 298 K): δ [ppm] = 6.80 (m {br}, 60 H; H^{para}), 6.40-6.60 (m {br}, 240 H; H^{meta}, H^{ortho}), 5.10 (s {br}, 120 H; CH_2), 4.84 (s {br}, 120 H; CH_2), 4.67 (s {br}, 120 H; CH_2)

^{31}P{^{1}H} NMR (CD_2Cl_2, 400 MHz, 300 K): δ [ppm] = 140 (s {br}), 90 (s {br}), 79 (s {br})

Elemental analysis: Calculated (%) for $(C_{40}H_{35}FeP_5)_{12}(CuBr)_{18}(CH_2Cl_2)$ (11384 g/mol): C 50.75, H 3.74; found: C 50.99, H 3.75

1.5 Synthesis of [{$Cp^{Bn}Fe(\mu_6$-η^5:η^1:η^1:η^1:η^1:η^1-P_5)}$_{12}${CuBr}$_{51}${CH_3CN}$_8$] (3)

In a schlenk tube complex **1** (100 mg, 0.14 mmol) was dissolved in CH_2Cl_2 (25 mL) to give an intensive green solution and added to a colorless solution of CuBr (110 mg, 0.78 mmol) in 4 mL CH_3CN. The reaction mixture immediately turns red-brown and was stirred for two further hours. Afterwards first 2 mL of a solvent mixture of CH_2Cl_2/CH_3CN (2:1) and subsequently 20 mL of toluene were layered on top of it. The reaction mixture was allowed to stand in an undisturbed area at r.t.. Within two days the crystallization process starts with the formation of small red rods of **3** at the phase boundary. After complete diffusion the almost completely discoloured mother liquor decanted, the crystals were washed with hexane (4 x 5 mL) and dried under vacuum at r.t. to afford 180 mg (0.011 mmol, 96%) of **3**.

A higher molar ratio of CuBr (e.g. 6 eq.; 120 mg) referred to **1** leads to the crystallization of a small amount of CuBr besides **3**, a lower molar ratio of CuBr (e.g. 5 eq.; 100 mg) at the other hand leads to the crystallization of **2** besides **3**. Therefore, the molar ratio of 5.5 equivalents CuBr (110 mg) proved to be the ideal stoichiometry for the selective crystallization of **3**.

Analytical data of [{$Cp^{Bn}Fe(\mu_6$-η^5:η^1:η^1:η^1:η^1:η^1-P_5)}$_{12}${CuBr}$_{51}${CH_3CN}$_8$] (**3**):

Yield: 180 mg (0.011 mmol, 96%)

^{1}H NMR (CD_2Cl_2, 600 MHz, 298 K): δ [ppm] = 6.89 (m {br}, 60 H, H^{para}), 6.76 (m {br}, 120 H, H^{meta}), 6.63 (m {br}, 120 H, H^{ortho}), 4.57 (m, {br}, 120 H, CH_2)

^{31}P{^{1}H} NMR (CD_2Cl_2, 400 MHz, 300 K): δ [ppm] = 59.8 (s, {br})

Elemental analysis: Calculated (%) for $(C_{40}H_{35}FeP_5)_{12}(CuBr)_{51}(CH_3CN)_3$: (16156 g/mol): C 36.13, H 2.68, Br 25.2, Cu 20.0, Fe 4.15, N 0.26, P 11.50; found: C 35.94, H 2.80, Br 25.0, Cu 19.5, Fe 4.13, P 10.71.

2. X-ray Structure Analysis

2.1 Data Collection and Refinement

The diffraction data for structures **2-3** were collected at 123 K on an Agilent Technologies Gemini R-Ultra (**2b-Cl** and **2b-Br**), SuperNova CCD diffractometer equipped with Atlas detector (**2c-Cl**, **2c-Br**, **2d-Cl** and **2d-Br** and $(FeCp_2)$@**2-Cl** and SuperNova CCD diffractometer equipped with Atlas S2 (**2a-Cl** and $(FeCp_2)$@**2-Br)** or Titan S2 (**2a-Br** and **3**) detector, with CuK_α radiation (λ = 1.54178Å) using ω scans of 1° (0.5° in the case of **3**) frames. Absorption corrections were applied analytically from crystal faces using CrysAlisPro software. The structure **2b** was solved by direct methods and refined by full-matrix least-squares method against $|F|^2$ in anisotropic approximation using SHELX97 programs set (see Table S). The structures **2a-d** were found to be isostructural and therefore were refined using the earlier obtained structural model. All non-hydrogen atoms were refined anisotropically. Hydrogen atoms were refined as riding on pivot atoms.

The all variety of isostructural compounds **2a-d** (Table S 1) crystallize in $Pm\bar{3}n$ space group and contain two supramolecules per unit cell (Z=2) (Table S 2, Table S 3). The supramolecule lies in the Wykoff position *2a* with $m\bar{3}$ (T_d) symmetry; the independent part of it comprises half of the $Cp^{Bn}Fe(\eta^5\text{-}P_5)$ complex and 2 CuX units, Cu1, X1 atoms in Wykoff special position *16i* with {x, x, x} orbit, and Cu2 and X2 atoms in the Wykoff special position *24k* {0, y, z} orbit. In **2a** both CuX positions are fully occupied. Vacancy in the supramolecule **2b-d** is always found in one of two independent positions of CuX unit and was refined as follows. The Cu1 and X1 positions are always fully occupied with respect to their a.d.p. parameters. In many cases the Cu2 and X2 positions seem to be partly vacant since the atoms have higher a.d.p. parameters. The occupancies for ‘position 2’ were refined with their isotropic a.d.p. parameters being equal (EADP instruction in SHELX). As the X2 position is terminal and therefore can have slightly higher a.d.p., the refined occupancies were fixed at the resulting value for Cu2 atom. The constraint on Cu and X a.d.p.’s was then released and normal refinement procedure in anisotropic approximation was performed. Standard deviation in occupancies of Cu2 and X2 atoms and therefore the accuracy of vacant-to-complete supramolecule ratio was found as

follows. The occupancy factors for atoms in the position 2 were released to refine, and one refinement cycle was performed (L. S. = 1) with DAMP 0 0 instructions in SHELX. The resulting accuracy of composition was calculated as e.s.d.(Cu2)×12 according to the number of Cu2 positions in the supramolecule (Table S 1). Due to high crystallographic symmetry, the phenyl groups of Cp^{Bn} ligands are positionally disordered. Some of them were modeled as ideal hexagons with AFIX 66 and refined with equivalent a.d.p. parameters.

The guest molecules, DCM or ferrocene, in the inner cavity of the supramolecules are disordered. Slight rearrangement of occupancy factors on two independent but overlapping C/Cl positions for the encapsulated CH_2Cl_2 molecule (disordered total over 32 positions) slightly varies from structure to structure giving in sum a fully occupied position of the guest molecule. The ferrocene molecules in ($FeCp_2$)@**2-Cl** and ($FeCp_2$)@**2-Br** are disordered over 6 positions generated by symmetry operations with iron atom being in the center. Position of the iron atom (Wykoff position *2a*) was found to be fully occupied and was refined anisotropically. The carbon atoms were located from electron density map taking into account expected geometry and crystallographic disorder, and were refined in isotropic approximation with calculated occupancies to give 10 carbon atoms per 1 Fe atom. According to the high a.d.p's for the carbon atoms, there is a possibility that not all disordered positions for light atoms are found. However, no additional electron density on the ferrocene molecule was found. The crystal structure contains voids in the packing of supramolecules of about 1500 Å^3 per unit cell, in which single electron density peaks are found that however do not allow to locate any solvent molecule. The estimation based on residual electron density made with SQUEEZE/PLATON allows assigning the non-localized solvent portion as 4 C_7H_8+CH_2Cl_2 or 5 CH_2Cl_2 per unit cell, but still seems to be insufficient to fill such an accessible volume.

Despite the crystals of **3** are relatively large, the diffraction pattern fades quickly at d_{hkl}<1 Å. Therefore only data with $d_{hkl} \geq 0.9$ Å were used in thw structure refinement. The supramolecules **3** occupy special position 16e (Wyckoff notation) of space group *Fddd* with local symmetry 2, namely, the 2-fold axis parallel to *a* parameter. The presence of the 2-fold axis results in some crystallographic disorder. The structure refinement faced various problems due to relatively low orthorhombic symmetry resulted in 102 crystallographically independent heavy atoms, severe disorder of Cp^{Bn} ligands accompanied by partial disorder of Cu and Br atoms of the inorganic core, and luck of observed high-angle reflections. At the preliminary stages the

refinement was performed using Konnert-Hendrickson conjugate-gradient algorithm (CGLS instruction in SHELXL97 program), and only the final model was refined by full-matrix least-squares method. The occupancy factors for the Cu(I) dimers disordered over the 2-fold axis were set as 0.5. The occupancy factors for $Cu(NCMe)_2$ groups were set to 0.25 according to thermal parameters of the Cu atoms and to the presence of short intermolecular Me…Me and Me…Ph contacts that are inconsistent with mutual co-existing of $Cu(NCMe)_2$ groups in neighboring supramolecules. The occupancy factors of internal {Cu(NCMe)Br} groups were set to 0.25 according to the sterical requirements. The occupancy factors for Br atoms were determined after refinement with U_{iso} fixed at 0.05 $Å^{-1}$ level. Final occupancy factors for the Br atoms were set using information on chemical composition and charge balance requirements. During the final refinement all Cu, Br, Fe and P atoms were refined anisotropically with fixed occupancy factors. The carbon atoms of the Cp rings were refined anisotropically. The positions of the CH_2Ph fragments and NCMe ligands were located from $\Delta\rho$ maps and refined with some geometrical constraints (CMe) or in rigid body approximation (Ph groups) with U_{iso} of C atoms of Ph rings at 0.1 $Å^{-1}$ level. No any solvent molecules were located.

Packing motifs were analyzed with TOPOS 4.0 Professional program suit for crystal chemical analysis[2]. Bond lengths and bond angles are summarized in Table S 4 (for **2**) and Table S 5 (for **3**). CCDC-1019930 (compound **2a-Br**), -1019931 (compound **2b-Br**), -1019932 (compound **2c-Br**), -1019933 (compound **2d-Br**), -1019934 (compound **3**), -1019935 (compound $(FeCp_2)$@**2-Br**), -1019936 (compound **2b-Cl**), -1019937 (compound **2c'-Cl**), -1019938 (compound **2c-Cl**), -1019939 (compound **2d-Cl**) and, -1019940 (compound $(FeCp_2)$@**2-Cl**), contain the supplementary crystallographic data for this publication. These data can be obtained free of charge at www.ccdc.cam.ac.uk/conts/retrieving.html (or from the Cambridge Crystallographic Data Centre, 12 Union Road, Cambridge CB2 1EZ, UK; Fax: + 44-1223-336-033; e-mail: deposit@ccdc.cam.ac.uk).

Table S 1. The crystal structures of 80-vertex Cp^{Bn}-based supramolecules

Name	Formula	*20-n*	*a*, Å	V, Å^3	R_1
a) CH_2Cl_2@Supramolecules					
n = 0					
**2a-Br**	$(CH_2Cl_2)@[(Cp^{bz}FeP_5)_{12}(Cu\mathbf{Br})_{20}]\cdot4.8C_7H_8$	**20**	31.8316(3)	32253.4(5)	0.0997
1.5 > n > 1.2					
**2b-Cl***	$(CH_2Cl_2)@[(Cp^{bz}FeP_5)_{12}(Cu\mathbf{Cl})_{18.80(6)}]\cdot4.8C_7H_8$	**18.80(7)**	31.5993(2)	31552.4(3)	0.1028
**2b-Br**	$(CH_2Cl_2)@[(Cp^{bz}FeP_5)_{12}(Cu\mathbf{Br})_{18.54(4)}]\cdot4.8C_7H_8$	**18.53(4)**	31.7096(2)	31884.0(3)	0.0877
3.0 > n > 1.5					
**2c-Cl***	$(CH_2Cl_2)@[(Cp^{bz}FeP_5)_{12}(Cu\mathbf{Cl})_{17.00(7)}]\cdot4.8C_7H_8$	**17.00(7)**	31.6467(3)	31694.6(3)	0.1008
**2c'-Cl***	$(CH_2Cl_2)@[(Cp^{bz}FeP_5)_{12}(Cu\mathbf{Cl})_{17.60(6)}]\cdot4.8C_7H_8$	**17.60(6)**	31.6106(2)	31586.26(17)	0.0921
**2c-Br**	$(CH_2Cl_2)@[(Cp^{bz}FeP_5)_{12}(Cu\mathbf{Br})_{18.44(5)}]\cdot4.8C_7H_8$	**18.44(5)**	31.6059(3)	31572.2(5)	0.0900
4.8 > n > 3.0					
**2d-Cl***	$(CH_2Cl_2)@[(Cp^{bz}FeP_5)_{12}(Cu\mathbf{Cl})_{15.20(4)}]\cdot4.8C_7H_8$	**15.20(4)**	31.5997(5)	31553.6(9)	0.1146
**2d-Br**	$(CH_2Cl_2)@[(Cp^{bz}FeP_5)_{12}(Cu\mathbf{Br})_{15.59(6)}]\cdot4.8C_7H_8$	**15.59(6)**	31.71380(10)	31896.63(17)	0.0945
b) Ferrocene@Supramolecules					
1.5 > n > 1.2					
$(FeCp_2)$@**2-Cl**	$(FeCp_2)@[(Cp^{bz}FeP_5)_{12}(Cu\mathbf{Cl})_{18.68(6)}]\cdot4.8C_7H_8$	**18.68(6)**	31.52725(8)	31337.05(14)	0.1024
$(FeCp_2)$@**2-Br**	$(FeCp_2)@[(Cp^{bz}FeP_5)_{12}(Cu\mathbf{Br})_{18.56(3)}]\cdot4.8C_7H_8$	**18.56(3)**	31.6937(2)	31836.0(3)	0.0809

* Refined with the same model as Br derivatives

Table S 2. Crystallographic data on structure measurement and refinement for **2a-d** and **3**.

Compound	**2b-Cl**	**2c-Cl**	**2c'-Cl**	**2d-Cl**	$(FeCp_2)$@**2-Cl**
**Crystal data**					
Chemical formula	$C_{514.60}H_{460.40}Cl_{20.80}Cu_{18.80}Fe_{12}P_{60}$	$C_{514.60}H_{460.40}Cl_{19}Cu_{17}Fe_{12}P_{60}$	$CH_2Cl_2 \cdot C_{480}H_{420}Cl_{17.60}Cu_{17.60}Fe_{12}P_{60} \cdot 4.8(C_7H_8)$	$C_{514.60}H_{460.40}Cl_{17.20}Cu_{15.20}Fe_{12}P_{60}$	$C_{10}H_{10}Fe \cdot C_{480}H_{420}Cl_{18.68}Cu_{18.68}Fe_{12}P_{60} \cdot 4.8(C_7H_8)$
M_r	11104.74	10926.56	10985.60	10748.38	11193.97
Crystal system, space group	Cubic, $Pm\overline{3}n$	Cubic, $Pm\overline{3}n$	Cubic, $Pm\overline{3}n$	Cubic, $Pm\overline{3}n$	Cubic, $Pm\overline{3}n$
Temperature (K)	123	123	123	123	123
a (Å)	31.6285 (3)	31.6467 (2)	31.6106 (1)	31.5997 (5)	31.5272 (1)
V (Å^3)	31639.9 (5)	31694.6 (3)	31586.26 (17)	31553.6 (9)	31336.91 (17)
Z	2	2	2	2	2
Radiation type	Cu $K\alpha$	Cu $K\alpha$	Cu $K\alpha$	Cu $K\alpha$	Cu $K\alpha$
μ (mm^{-1})	5.36	5.22	5.38	5.11	5.51
Crystal size (mm)	0.47 × 0.39 × 0.26	0.29 × 0.23 × 0.07	0.21 × 0.16 × 0.10	0.16 × 0.12 × 0.09	0.38 × 0.28 × 0.24
**Data collection**					
Diffractometer	Xcalibur, Ruby, Gemini ultra diffractometer	SuperNova, Single source at offset, Atlas diffractometer	SuperNova, Single source at offset, Atlas diffractometer	SuperNova, Single source at offset, Atlas diffractometer	SuperNova, Single source at offset, Atlas diffractometer
Absorption correction	Analytical	Analytical	Analytical	Analytical	Analytical
T_{min}, T_{max}	0.222, 0.393	0.420, 0.678	0.536, 0.664	0.579, 0.696	0.262, 0.418
No. of measured, independent and observed [$I > 2\sigma(I)$] reflections	23700, 5298, 3626	21973, 5709, 4242	22450, 5476, 4164	21910, 5707, 4554	163634, 5693, 4708
R_{int}	0.047	0.030	0.027	0.049	0.042
(sin θ/λ)$_{max}$ (Å^{-1})	0.613	0.631	0.624	0.631	0.627
**Refinement**					
$R[F^2 > 2\sigma(F^2)]$, $wR(F^2)$, S	0.103, 0.302, 1.95	0.101, 0.302, 1.06	0.092, 0.297, 1.21	0.116, 0.355, 1.50	0.102, 0.315, 2.59
No. of reflections	5298	5709	5476	5707	5693
No. of parameters	211	198	198	228	209
No. of restraints	5	5	5	6	8

H-atom treatment	H-atom parameters constrained	H-atom parameters constrained	H-atom parameters constrained	H atoms treated by a mixture of independent and constrained refinement	H atoms treated by a mixture of independent and constrained refinement
$\Delta\rangle_{max}$, $\Delta\rangle_{min}$ (e Å^{-3})	3.18, -0.96	2.46, -1.00	2.62, -1.08	3.29, -0.91	3.26, -1.18

Table S 3. Crystallographic data on structure measurement and refinement for **2a-d** and **3** (*continued*).

Compound	**2a-Br**	**2b-Br**	**2c-Br**	**2d-Br**	($FeCp_2$)@**2-Br**	**3**
**Crystal data**						
Chemical formula	$C_{514.60}H_{460.40}Br_{20}Cl_2Cu_{20}Fe_{12}P_{60}$	$C_{514.60}H_{460.40}Br_{18.53}Cl_2Cu_{18.53}Fe_{12}P_{60}$	$C_{514.60}H_{460.40}Br_{18.44}Cl_2Cu_{18.44}Fe_{12}P_{60}$	$C_{514.60}H_{460.40}Br_{15.59}Cl_2Cu_{15.59}Fe_{12}P_{60}$	$C_{523.60}H_{468.40}Br_{18.56}Cu_{18.56}Fe_{13}P_{60}$	$C_{496}H_{444}Br_{51}Cu_{51}Fe_{12}N_8P_{60}$
M_r	12112.73	11902.58	11888.95	11479.40	12007.27	16360.94
Crystal system, space group	Cubic, $Pm\overline{3}n$	Cubic, $Pm\overline{3}n$	Cubic, $Pm\overline{3}n$	Cubic, $Pm\overline{3}n$	Cubic, $Pm\overline{3}n$	orthorhombic, *Fddd*
Temperature (K)	123	123	123	123	123	123
a (Å)	31.8316(3)	31.7096(2)	31.6059(3)	31.7138(1)	31.6937(2)	61.8752(5)
b (Å)	31.8316(3)	31.7096(2)	31.6059(3)	31.7138(1)	31.6937(2)	68.8668(5)
c (Å)	31.8316(3)	31.7096(2)	31.6059(3)	31.7138(1)	31.6937(2)	69.6595(5)
V (Å^3)	32253.4(5)	31884.0(3)	31572.2(5)	31896.63(17)	31836.0(3)	296829(4)
Z	2	2	2	2	2	
Radiation type	Cu *K*α	Cu *K*α	Cu *K*α	Cu *K*α	Cu *K*α	Cu *K*α
μ (mm^{-1})	6.04	5.95	6.00	5.62	6.06	8.06
Crystal size (mm)	0.10 × 0.10 × 0.06	0.25 × 0.18 × 0.10	0.25 × 0.21 × 0.17	0.30 × 0.25 × 0.21	0.20 × 0.17 × 0.13	0.56 × 0.17 × 0.12
**Data collection**						
Diffractometer	SuperNova, Single source at offset, TitanS2 diffractometer	Xcalibur, Ruby, Gemini ultra diffractometer	SuperNova, Single source at offset, Atlas diffractometer	SuperNova, Single source at offset, Atlas diffractometer	SuperNova, Single source at offset, AtlasS2 diffractometer	SuperNova, Single source at offset, TitanS2 diffractometer
Absorption correction	Analytical	Analytical	Analytical	Analytical	Analytical	Analytical
T_{min}, T_{max}	0.439, 0.575	0.375, 0.614	0.334, 0.503	0.340, 0.462	0.250, 0.412	0.188, 0.472
No. of measured, independent and observed [$I > 2\sigma(I)$] reflections	29869, 5696, 2818	42583, 5182, 2968	20680, 5492, 3813	84734, 5850, 4442	90294, 5631, 3546	129123, 52924, 32114
R_{int}	0.0817	0.056	0.041	0.035	0.050	0.035
$(\sin \theta/\lambda)_{max}$ (Å^{-1})	0.613	0.605	0.623	0.630	0.622	0.555
**Refinement**						
$R[F^2 > 2\sigma(F^2)]$, $wR(F^2)$, S	0.100, 0.311, 0.96	0.088, 0.284, 0.97	0.090, 0.287, 1.11	0.095, 0.307, 1.27	0.081, 0.264, 0.99	0.155, 0.453, 2.25
No. of reflections	5696	5182	5492	5850	5631	52924

No. of parameters	198	209	209	202	209	1412
No. of restraints	5	5	6	5	6	55
H-atom treatment	H atoms treated by a mixture of independent and constrained refinement	H atoms treated by a mixture of independent and constrained refinement	H-atom parameters constrained	H-atom parameters constrained	H atoms treated by a mixture of independent and constrained refinement	H atoms treated by a mixture of independent and constrained refinement
$\Delta\rangle_{max}$, $\Delta\rangle_{min}$ (e Å^{-3})	2.13, -0.97	1.46, -0.97	2.72, -1.02	2.74, -0.95	1.40, -0.87	6.367, -2.741

Computer programs: *CrysAlis PRO*, Agilent Technologies, Version 1.171.36.28 (release 01-02-2013 CrysAlis171.NET), *SHELXS97* (Sheldrick, 2008), *SHELXL97* (Sheldrick, 2008).

2.2 Selected Bond distances

Table S 4. Selected bond distances in **2-Br** and **2-Cl.**

Compound	**2b-Cl**	**2c-Cl**	**2c'-Cl**	**2d-Cl**	($FeCp_2$)@ **2-Cl**	**2a-Br**	**2b-Br**	**2c-Br**	**2d-Br**	($FeCp_2$)@ **2-Br**
Nature of **X**	**Cl**					**Br**				
Cu1—**X**1	2.191(4)	2.193(3)	2.195(2)	2.205(3)	2.192(3)	2.323(3)	2.320(2)	2.3148(19)	2.3343(17)	2.3173(18)
Cu2—**X**2	2.195(3)	2.205(3)	2.207(3)	2.229(4)	2.196(2)	2.334(2)	2.325(2)	2.3206(17)	2.349(2)	2.3233(17)
Cu2—P1	2.301(3)	2.299(2)	2.301(2)	2.304(3)	2.302(2)	2.305(4)	2.300(3)	2.300(3)	2.307(3)	2.299(3)
Cu1—P2 ×3	2.291(2)	2.2960(15)	2.2968(14)	2.2970(15)	2.2936(15)	2.294(3)	2.294(2)	2.2890(17)	2.2918(15)	2.2907(17)
Cu2—P3 ×2	2.292(2)	2.2896(17)	2.2911(15)	2.2803(18)	2.2911(16)	2.302(3)	2.297(2)	2.2925(18)	2.2807(17)	2.2935(19)
Fe1—C1 ×2	2.092(7)	2.097(6)	2.097(5)	2.084(6)	2.100(6)	2.092(9)	2.089(7)	2.088(6)	2.092(5)	2.096(6)
Fe1—C2 ×2	2.086(8)	2.098(6)	2.092(5)	2.105(6)	2.101(6)	2.113(9)	2.079(7)	2.095(6)	2.097(5)	2.094(6)
Fe1—C3	2.075(11)	2.099(8)	2.106(8)	2.089(8)	2.109(8)	2.090(13)	2.085(11)	2.093(9)	2.095(7)	2.103(9)
Fe1—P1	2.372(3)	2.374(2)	2.374(2)	2.379(2)	2.372(2)	2.381(4)	2.379(3)	2.372(3)	2.383(2)	2.377(3)
Fe1—P2 ×2	2.359(2)	2.3594(16)	2.3597(15)	2.3602(15)	2.3610(16)	2.372(3)	2.365(2)	2.3605(18)	2.3642(15)	2.3629(18)
Fe1—P3 ×2	2.370(2)	2.3755(18)	2.3744(16)	2.3770(17)	2.3734(17)	2.381(3)	2.387(2)	2.375(2)	2.3828(17)	2.385(2)
P1—P2 ×2	2.099(2)	2.1024(18)	2.1010(17)	2.1071(17)	2.0998(18)	2.100(3)	2.100(3)	2.097(2)	2.1024(17)	2.101(2)
P2—P3	2.098(3)	2.100(2)	2.1009(19)	2.1039(19)	2.100(2)	2.094(3)	2.103(3)	2.101(2)	2.1077(19)	2.105(2)
P3—P3	2.092(4)	2.098(3)	2.099(3)	2.100(3)	2.101(3)	2.092(5)	2.095(4)	2.093(3)	2.103(3)	2.095(3)

Table S 5. Geometric parameters of the heavy core in **3**.

**P—P**	Bond, Å	**Fe—P**	Bond, Å	**Fe—C**	Bond, Å	**Cu—P**	Bond, Å	**Cu—Br**	Bond,**Å**	**Cu—Br**	Bond, Å
P11—P12	2.126(10)	Fe1—P11	2.395(8)	Fe1—C11	2.16(3)	Cu3—P53	2.223(8)	Cu1B—Br1	2.47(2)	Cu14—Br16	2.561(5)
P11—P15	2.112(11)	Fe1—P12	2.394(8)	Fe1—C12	2.11(3)	Cu4B—P45	2.262(14)	Cu1B—Br2^{i}	2.362(17)	Cu14—Br17	2.427(6)
P12—P13	2.117(10)	Fe1—P13	2.363(8)	Fe1—C13	2.12(3)	Cu4B—P51^{i}	2.238(14)	Cu1A—Br1^{i}	2.49(3)	Cu15—Br9	2.510(5)
P13—P14	2.086(10)	Fe1—P14	2.392(9)	Fe1—C14	2.12(3)	Cu4A—P45	2.274(12)	Cu1A—Br2	2.635(10)	Cu15—Br16	2.587(5)
P14—P15	2.091(10)	Fe1—P15	2.367(9)	Fe1—C15	2.10(3)	Cu4A—P51^{i}	2.265(13)	Cu1A—Br3	2.475(9)	Cu15—Br17	2.411(5)
P21—P22	2.091(10)	Fe2—P21	2.373(7)	Fe2—C21	2.11(2)	Cu5—P55^{i}	2.260(8)	Cu2B—Br27	2.750(12)	Cu16—Br16	2.423(5)
P21—P25	2.102(10)	Fe2—P22	2.382(9)	Fe2—C22	2.08(3)	Cu5—P63^{i}	2.261(8)	Cu2B—Br28	2.368(9)	Cu16—Br18	2.669(5)
P22—P23	2.097(10)	Fe2—P23	2.367(8)	Fe2—C23	2.04(3)	Cu7—P44	2.281(9)	Cu2A—Br28	2.314(8)	Cu16—Br20	2.394(5)
P23—P24	2.098(13)	Fe2—P24	2.384(8)	Fe2—C24	2.08(3)	Cu7—P64^{i}	2.264(9)	Cu2A—Br29	2.694(12)	Cu17—Br18	2.667(5)
P24—P25	2.096(11)	Fe2—P25	2.375(8)	Fe2—C25	2.10(3)	Cu8—P42	2.217(8)	Cu2—Br2	2.544(6)	Cu17—Br19	2.422(5)
P31—P32	2.113(11)	Fe3—P31	2.395(8)	Fe3—C31	2.14(3)	Cu9—P32	2.224(9)	Cu2—Br3	2.465(7)	Cu17—Br20	2.410(5)
P31—P35	2.119(10)	Fe3—P32	2.380(9)	Fe3—C32	2.07(3)	Cu10—P12	2.243(9)	Cu2—Br4	2.372(7)	Cu18—Br18	2.597(5)
P32—P33	2.082(10)	Fe3—P33	2.360(8)	Fe3—C33	2.08(3)	Cu11—P43	2.245(9)	Cu3—Br2	2.608(6)	Cu18—Br19	2.422(6)
P33—P34	2.090(10)	Fe3—P34	2.379(7)	Fe3—C34	2.15(3)	Cu12—P65^{i}	2.215(9)	Cu3—Br4	2.431(5)	Cu18—Br21	2.402(6)
P34—P35	2.113(10)	Fe3—P35	2.360(8)	Fe3—C35	2.04(2)	Cu13—P11	2.216(8)	Cu3—Br9	2.478(5)	Cu19—Br18	2.681(5)
P41—P42	2.098(11)	Fe4—P41	2.365(9)	Fe4—C41	2.14(3)	Cu14—P54	2.208(9)	Cu4B—Br5	2.279(11)	Cu19—Br21	2.397(5)
P41—P45	2.108(11)	Fe4—P42	2.398(8)	Fe4—C42	2.11(3)	Cu15—P31	2.235(8)	Cu4B—Br6	2.818(16)	Cu19—Br22	2.450(5)
P42—P43	2.141(11)	Fe4—P43	2.385(8)	Fe4—C43	2.11(3)	Cu16—P62	2.209(9)	Cu4A—Br3	2.662(13)	Cu20—Br22	2.477(5)
P43—P44	2.103(11)	Fe4—P44	2.350(9)	Fe4—C44	2.17(3)	Cu17—P35	2.234(9)	Cu4A—Br5	2.337(10)	Cu20—Br23	2.404(5)
P44—P45	2.085(10)	Fe4—P45	2.399(10)	Fe4—C45	2.13(3)	Cu18—P25	2.213(9)	Cu5—Br6	2.600(7)	Cu20—Br25	2.567(5)
P51—P52	2.094(10)	Fe5—P51	2.366(9)	Fe5—C51	2.11(3)	Cu19—P61	2.215(8)	Cu5—Br7	2.332(6)	Cu21—Br23	2.421(6)
P51—P55	2.103(11)	Fe5—P52	2.383(9)	Fe5—C52	2.09(3)	Cu20—P24	2.216(8)	Cu6—Br5	2.509(18)	Cu21—Br25	2.601(5)
P52—P53	2.102(11)	Fe5—P53	2.398(8)	Fe5—C53	2.09(3)	Cu21—P15^{i}	2.211(8)	Cu6—Br6	2.529(12)	Cu21—Br26	2.364(9)
P53—P54	2.090(11)	Fe5—P54	2.358(8)	Fe5—C54	2.10(3)	Cu26—P14	2.258(9)	Cu7—Br6	2.502(7)	Cu22—Br24	2.029(17)
P54—P55	2.097(11)	Fe5—P55	2.387(8)	Fe5—C55	2.12(3)	Cu26—P22	2.263(9)	Cu7—Br8	2.372(15)	Cu22—Br24^{i}	2.409(17)
P61—P62	2.119(10)	Fe6—P61	2.400(8)	Fe6—C61	2.10(3)	Cu27—P21	2.271(8)	Cu8—Br9	2.480(5)	Cu22—Br25	2.744(9)
P61—P65	2.109(11)	Fe6—P62	2.356(8)	Fe6—C62	2.11(3)	Cu27—P34	2.265(7)	Cu8—Br10	2.560(5)	Cu22—Br26	2.414(11)
P62—P63	2.113(10)	Fe6—P63	2.398(8)	Fe6—C63	2.11(3)	Cu22—P23^{i}	2.374(12)	Cu8—Br11	2.442(5)	Cu23—Br24	2.558(11)
P63—P64	2.103(11)	Fe6—P64	2.353(9)	Fe6—C64	2.09(3)	Cu23—P23	2.125(10)	Cu9—Br10	2.660(6)	Cu23—Br25^{i}	2.360(9)
P64—P65	2.097(11)	Fe6—P65	2.384(9)	Fe6—C65	2.10(3)	P15—Cu21^{i}	2.211(8)	Cu9—Br11	2.375(6)	Cu23—Br25	2.830(10)
**Cu—Cu**	Bond, Å	**Cu—N**	Bond, Å	**Cu_{inner}—P**	Bond, Å	P23—Cu22^{i}	2.375(12)	Cu9—Br27	2.438(7)	Cu25—Br28	2.461(12)

Cu1A—Cu1B	2.84(2)	Cu6—N11	1.99(5)	Cu3I—P31	2.476(13)	P51—Cu4B^{i}	2.238(14)	Cu10—Br10	2.570(7)	Cu25—Br29	2.622(10)
Cu1A—Cu2	2.997(11)	Cu6—N12	1.99(4)	Cu3I—P32	2.424(12)	P51—Cu4A^{i}	2.265(13)	Cu10—Br12	2.350(7)	Cu23—Br24^{i}	2.377(10)
Cu2—Cu3	2.993(7)	Cu14—N31	2.06(4)	Cu4I—P61^{i}	2.439(13)	P52—Cu1A^{i}	2.283(11)	Cu10—Br27	2.520(7)	Cu26—Br29	2.598(6)
Cu8—Cu9	2.997(6)	Cu25—N21	1.94(6)	Cu4I—P65^{i}	2.482(13)	P55—Cu5^{i}	2.260(8)	Cu11—Br12	2.376(7)	Cu26—Br30	2.332(5)
Cu9—Cu10	3.009(8)	Cu25—N22	1.96(6)	Cu1I—P42	2.384(14)	P63—Cu5^{i}	2.261(8)	Cu11—Br13	2.431(7)	Cu27—Br29	2.489(6)
Cu11—Cu12	2.957(7)	Cu27—N41	1.90(3)	Cu1I—P43	2.426(14)	P64—Cu7^{i}	2.264(9)	Cu11—Br14	2.674(6)	Br1—Cu1A^{i}	2.49(3)
Cu14—Cu15	2.977(7)	**Cu$_{inner}$—Br$_{inner}$**	Bond, Å	Cu2I—P11	2.365(13)	P65—Cu12^{i}	2.215(9)	Cu12—Br13	2.405(6)	Br2—Cu1B^{i}	2.362(17)
Cu16—Cu17	2.747(6)	Cu1I—Br1I	2.446(14)	Cu2I—P12	2.422(13)	Cu1B—P52	2.166(19)	Cu12—Br14	2.685(6)	Br22—Cu13^{i}	2.466(6)
Cu18—Cu19	3.042(6)	Cu2I—Br2I	2.430(14)	P61—Cu4I^{i}	2.440(13)	Cu1A—P52^{i}	2.283(11)	Cu12—Br15	2.371(6)	Br24—Cu22^{i}	2.408(17)
Cu20—Cu21	3.008(6)	Cu3I—Br2I	2.505(13)	P65—Cu4I^{i}	2.482(13)	Cu2B—P13	2.284(11)	Cu13—Br14	2.590(5)	Br24—Cu23^{i}	2.377(10)
Cu22—Cu23	2.779(11)	Cu4I—Br1I	2.514(13)	**Cu$_{inner}$—N$_{inner}$**	Bond, Å	Cu2B—P33	2.273(12)	Cu13—Br15	2.414(6)	Br25—Cu23^{i}	2.360(9)
		Cu4I—Br14	2.802(12)	Cu2I—N2I	2.00(6)	Cu2A—P13	2.268(12)	Cu13—Br22^{i}	2.466(6)		
		Cu3I—Br10	2.749(12)	Cu3I—N3I	2.03(6)	Cu2—P41	2.209(9)				
				Cu4I—N4I	2.04(7)	Cu2A—P33	2.293(11)				
				Cu1I—N1I	2.05(6)						

Symmetry code(s): (i) *x*, -*y*+ 7/4, - *z*+ 7/4; (ii) -*x*+3/2, -*y*+3/2, -*z*+2.

2.3 Additional Figures of 2a-Br, $(FeCp_2)$@2-Br and 3

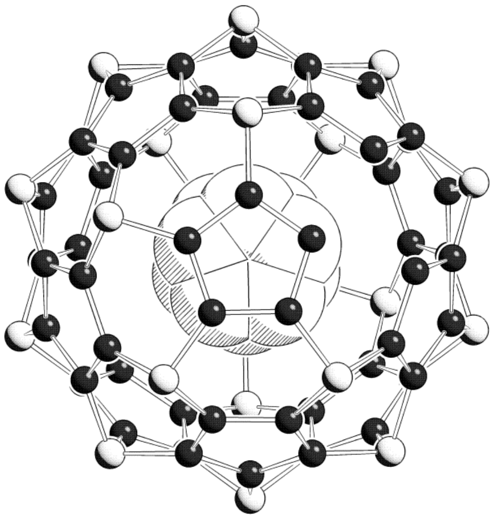

Fig. S1: Scaffold of the molecular structure of **$(FeCp_2)$@2-Br**. [Cp^{Bn}Fe] fragments, Br, and H atoms are omitted for clarity.

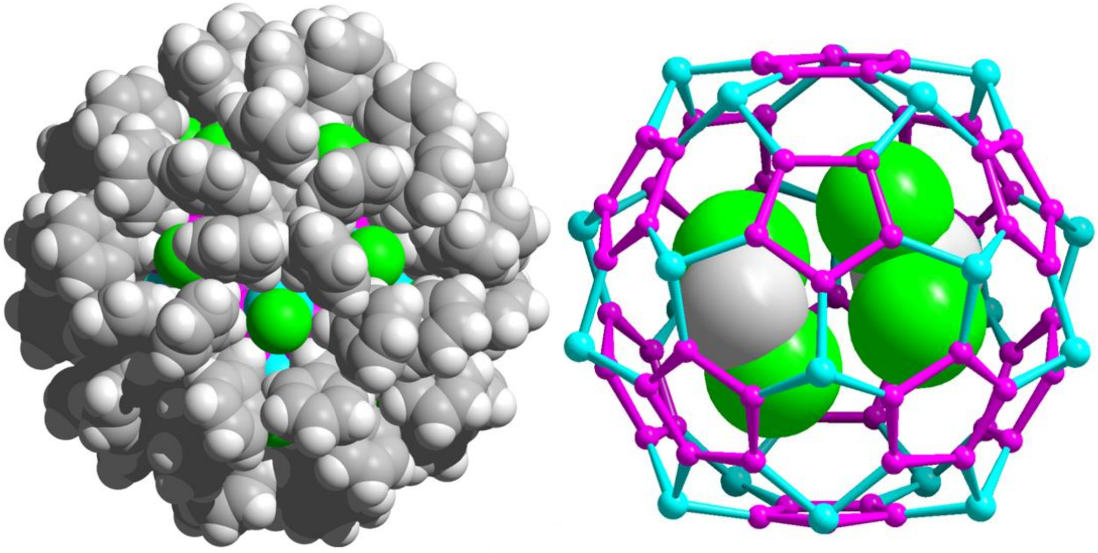

Fig. S 2. The supramolecule **2a-Br** (left, van der Waals spheres) and its inorganic scaffold (right) with two positions of the incorporated molecule CH_2Cl_2 (showed in van der Waals spheres).

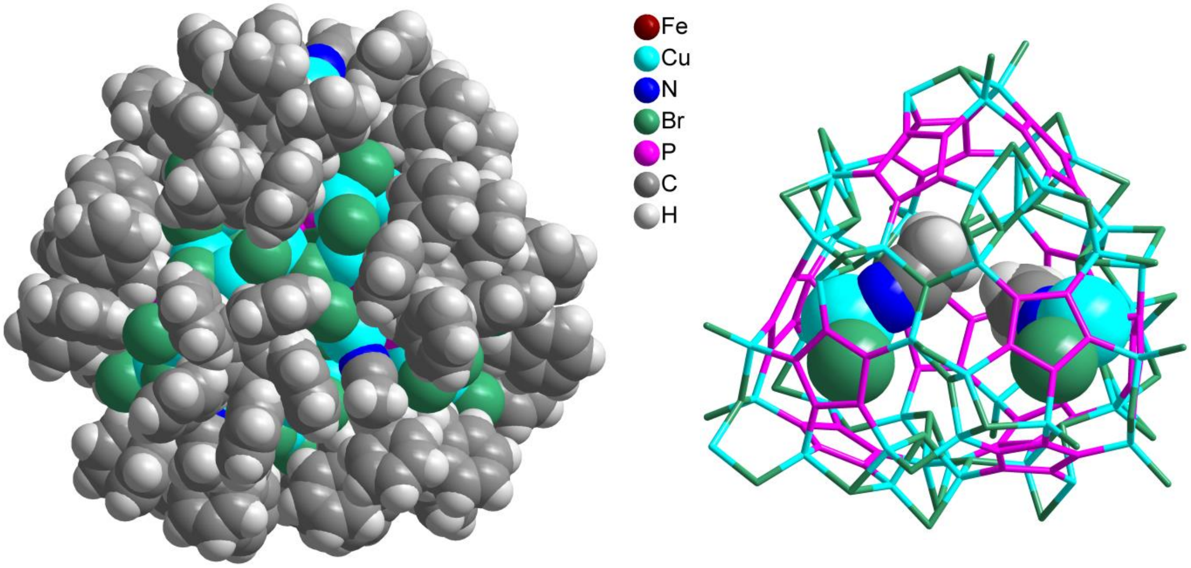

Fig. S 3. The supramolecule **3** (left, van der Waals spheres) and its inorganic scaffold (right) with two CuBr(NCMe) groups occupying the cavity (showed in van der Waals spheres).

3. NMR Experiments in Solution

NMR spectra were recorded on a Bruker Avance 400 MHz spectrometer equipped with a BBO probe with z-gradient and BVT 2000 temperature control unit at 300 K, a Bruker Avance III 600 MHz spectrometer equipped with a TBI $^{1}H/^{31}P$-BB z-gradient probe and BVT 3000 unit at 300 K and an Avance III 600 MHz spectrometer equipped with a 5 mm TCI z-gradient cryo

probe and BVT 3000 unit at 298 K. The spectra were processed with the Bruker program Topspin® and the diffusion coefficients were calculated with the Bruker software *T1/T2* package. All experimental diffusion coefficients were within a standard deviation of ±3 % and are stated as temperature- and viscosity-corrected diffusion coefficients in the manuscript.

For the calibration of the ^{31}P chemical shifts, the Ξ value corresponding to TMS was applied. For the calibration of the ^{1}H and ^{13}C chemical shifts and for the temperature- and viscosity-correction of the diffusion coefficients, TMS (tetramethylsilane) was added to each sample. All ^{1}H-diffusion measurements were performed with the convection suppressing DSTE (double stimulated echo) pulse sequence, developed by Mueller and Jerschow[4] in a pseudo 2D mode. For each experiment, 2 dummy scans and 16 scans were used with a relaxation delay of 2 s. Sinusoidal shapes were used for the gradients and a linear gradient ramp with either 12 or 16 increments between 5 and 95 % of the maximum gradient strength was applied for the diffusion relevant gradients. For the homospoil gradients, 7.046, 10.700, and 9.165 G cm^{-1} were applied for HS_1, HS_2, and HS_3. The length of the gradient pulse δ was adjusted for every species in the sample to achieve appropriate signal attenuation curves. As a result, a δ of 1.5 ms for TMS, 2.2 ms for the monomer and 3.2 ms for the supramolecules was used in most cases. A diffusion time Δ of 50 ms was used for all experiments.

Sample concentrations of 33 mM of **1** (15 mg in 0.6 mL solvent) in CD_2Cl_2 or CD_2Cl_2/CD_3CN mixtures were typically applied for the NMR measurements. The different samples were prepared by stirring of **1** together with CuX (X = Cl, Br) in the respective solvent or solvent mixture. After a reaction time of one hour each sample was filtrated and then characterized. Assignments of proton, carbon and phosphorous resonances of the species were obtained by one- and two-dimensional NMR spectra (^{1}H, ^{31}P, ^{1}H,^{13}C-HSQC, ^{1}H,^{13}C-HMBC, ^{1}H,^{1}H-ROESY and ^{1}H,^{1}H-NOESY (mixing times of 600, 350 and 100 ms) spectra (Fig. S 4).

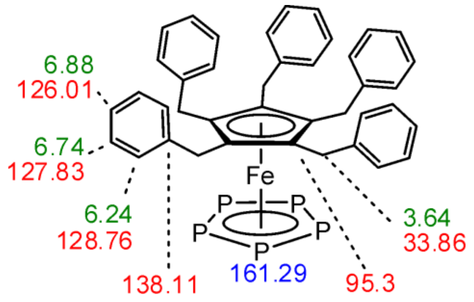

Fig. S 4. ^{1}H, ^{13}C and ^{31}P chemical shifts of **1** in CD_2Cl_2 at 298 K.

3.1 CuX (X = Cl, Br) titration in CD_2Cl_2 with 9 vol% CD_3CN

By changing the amount of CuX the formation trends of the different complexes in solution can be clearly followed. The experiment with CuBr is shown in Fig. S 5 and explained in more detail here, the one with CuCl is illustrated in Fig. S 6. As shown in Fig. S 5a, without CuBr only **1** was present in solution. Upon the addition of 0.5 CuBr (see Fig. S 5b), several other broad and overlapping signals in the ^{1}H spectrum (6.80 ppm for H_{para} and 6.40-6.60 ppm for H_{ortho} and H_{meta} and at least three signals at 5.10, 4.84 and 4.67 ppm for the methylene protons (data not shown)) were detected, which all showed the same ^{1}H diffusion coefficient (see Table S 7). In the ^{31}P spectrum, at least three very broad signals in the range of 65-125 ppm were observed, indicating P_5-rings with a varying CuBr coordination number. All those spectroscopic properties are in agreement with **2b-d.** A reliable differentiation between **2b-d** is not possible, however the ^{31}P chemical shift trend from **1** to **2a** suggests a high field shift with higher CuBr content and initiated by these NMR spectra the experimental conditions for the crystallization of **2b-d** were found. With one equivalent of CuBr (see Fig. S 5c), the amount of **2b-d** further increased at the expense of **1**. In addition, other species with separated ^{1}H chemical shifts for the meta and ortho protons were observed under the ^{1}H signals of **2b-d**. In the ^{31}P spectrum additionally a sharp signal at 63.80 ppm and broader signals at 49.8-57.9 ppm appeared which could be assigned to **2aCD$_2$Cl$_2$** and **2aCD$_3$CN**.

In the sample with 1.7 equivalents (see Fig. S 5d) the molar **1**:CuBr ratio was 12:20, the same ratio as in ideal **2a** crystals. Here, in the ^{1}H spectrum the amount of **1** and **2b-d** was drastically reduced and sharp signals with recognizable coupling fine structures at 6.75, 6.56, 6.44 and 5.09 ppm were detected. The very small linewidths of the ^{1}H-signals of **2aCD$_3$CN** are in agreement with the high symmetry of **2a**. The broader linewidths of **2aCD$_2$Cl$_2$** are most probably due to chemical exchange broadening with **2aCD$_3$CN** increased by the small amount of **2aCD$_2$Cl$_2$** and the tiny chemical shift deviation. In ^{1}H spectra in pure CD_2Cl_2 or with high amounts of **2aCD$_2$Cl$_2$** (see below) the linewidths are significantly smaller corroborating again the high symmetry of **2aCD$_2$Cl$_2$**. In the ^{31}P spectrum at 1.7 eq. of CuBr a sharp singlet at 63.80 ppm for **2aCD$_2$Cl$_2$** and a broader signal at 58.8 ppm for **2aCD$_3$CN** is detected. Here, the very

small linewidths of the ^{31}P signal of **2aCD$_2$Cl$_2$** indicates a defined coordination sphere identical for all ^{31}P species in **2aCD$_2$Cl$_2$**. In contrast the broad ^{31}P signal of **2aCD$_3$CN** suggests different fast exchanging ^{31}P environments possibly caused by partial exchange of Br and CD_3CN.

At 4 eq. of CuBr (see Fig. S 5e), the molar **1**:CuBr ratio was in the range of **3** (12:51). In the ^{1}H spectrum, the amount of **2aCD$_3$CN** and **2aCD$_2$Cl$_2$** was reduced and the signals of **3** appeared, whose aromatic signals (H_{para} 6.89 ppm, H_{meta} 6.78 ppm, H_{ortho} 6.63 ppm) were downfield shifted and the methylene signal (4.58 ppm) upfield shifted compared to **2b-d**, **2aCD$_2$Cl$_2$** and **2aCD$_3$CN**. In the ^{31}P spectrum no new signal appeared but a slight reduction of the ^{31}P chemical shift of **2aCD$_3$CN** indicates an overlap with **3**. With 8 eq. of CuBr (see Fig. S 5f), a higher amount of **3**, a lower amount of **2aCD$_3$CN** and only traces of **2aCD$_2$Cl$_2$** were observed (from the ^{1}H integration: 40 % **2aCD$_3$CN**, 4 % **2aCD$_2$Cl$_2$** and 56 % **3**).

The combined data from all NMR experiments indicate that **2aCD$_3$CN** and **2aCD$_2$Cl$_2$** are just different conformers of the same species, which are preferentially stabilized by the deviating solvent interactions in CD_3CN and CD_2Cl_2 and show a chemical exchange being slow on the NMR time scale. Solvent induced conformational changes are well known from peptide as well as protein chemistry and recently we extensively investigated the solvent dependent conformational preferences of acylguanidines and their complexes.[3] A crystal structure of **2aCD$_2$Cl$_2$** could not be obtained and the NMR spectra of **2aCD$_2$Cl$_2$** give only information about the composition and the size of **2aCD$_2$Cl$_2$** but not about the conformation. Therefore, any statement about the conformational differences between **2aCD$_3$CN** and **2aCD$_2$Cl$_2$** would be pure speculation.

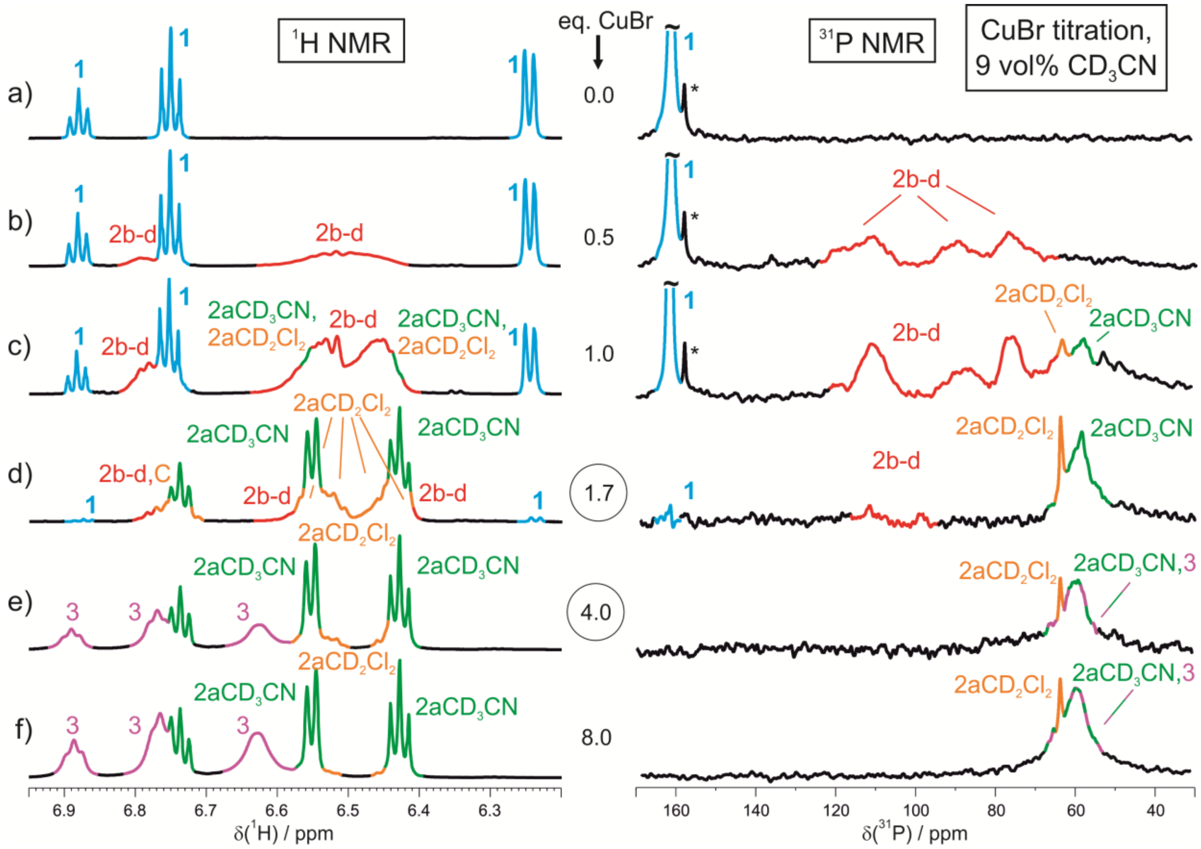

Fig. S 5. ^{1}H (left) and ^{31}P spectra (right) of samples containing **1** and increasing equivalents of CuBr: a) 0.0 eq., b) 0.5 eq., c) 1 eq., d) 1.7 eq., e) 4 eq., f) 8 eq., each in CD_2Cl_2 with 9 vol% CD_3CN. ^{1}H spectra at 298 K and 600 MHz, ^{31}P spectra at 300 K and 400 MHz.

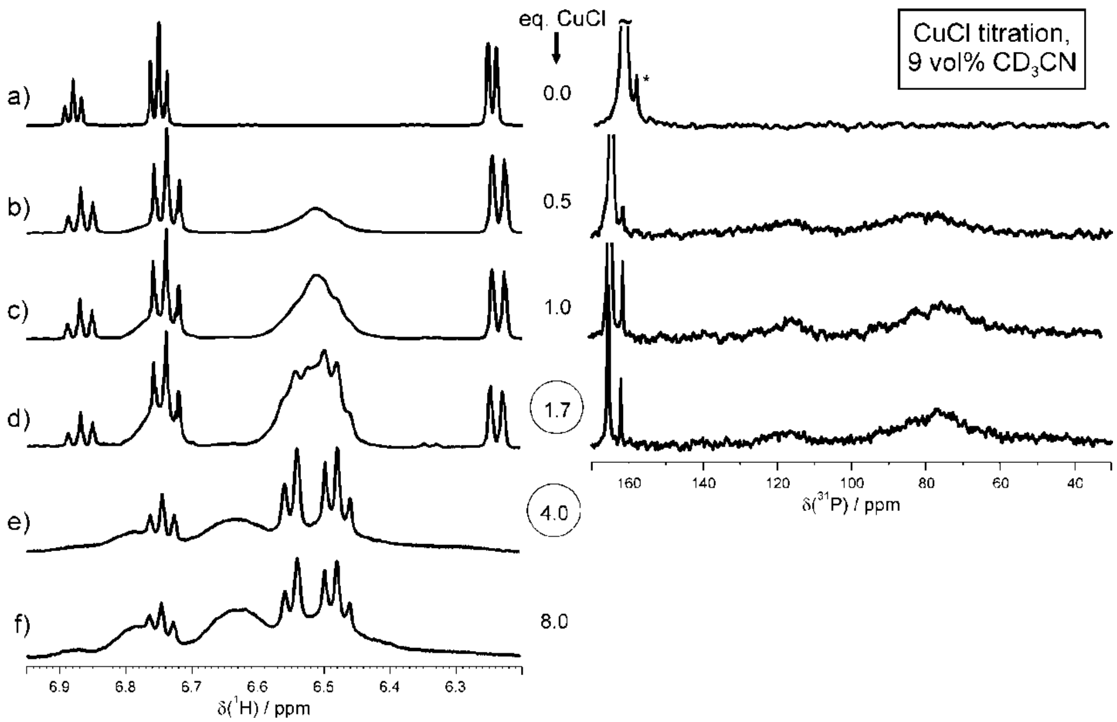

Fig. S 6. ^{1}H (left) and ^{31}P spectra (right) of samples containing **1** and increasing equivalents of CuCl: a) 0.0 eq., b) 0.5 eq., c) 1 eq., d) 1.7 eq., e) 4 eq., f) 8 eq., each in CD_2Cl_2 with 9 vol% CD_3CN at 300 K and 400 MHz.

3.2 Pure CD_2Cl_2: CuBr titration and dissolving crystals 2a-Br and 2b-d-Br

The spectra in pure CD_2Cl_2 confirm these assignments and especially the assignment of **2aCD2Cl2**. The solubility of CuBr in pure CD_2Cl_2 is quite low, therefore the samples were prepared by stirring a solution of **1** over solid CuBr for two hours or one, two, five and ten days and subsequent filtration (whereby removing CuBr stopped the reaction). The resulting ^{1}H and ^{31}P spectra are shown in Fig. S 7a-e. In case of the CuBr titration mainly the ^{31}P spectra are discussed due to the severe chemical shift overlap of the supramolecules in the ^{1}H spectra.

The low solubility of CuBr in pure CD_2Cl_2 is directly reflected in the slow formation of the supramolecules. After two hours (see Fig. S 7a), beside **1** only several species of **2b-d** were detected showing several subspecies in the range of 65-125 ppm in the ^{31}P spectrum. After one day (see Fig. S 7b), **1** was completely consumed. The distribution of the **2b-d** species is clearly shifted to those with higher copper contents (species at higher field increase at the expense of

those at lower field). In addition, the sharp signal of **2aCD$_2$Cl$_2$** appeared. After two days (see Fig. S 7c) the amount of **2aCD$_2$Cl$_2$** further increased at the expense of **2b-d** and a small signal C was detected, which could not be assigned so far. At longer reaction times (see Fig. S 7d and e), just a further small shift between **2b-d** and **2aCD$_2$Cl$_2$** was observed. C remained constant and **3** requiring small amounts of CD$_3$CN was not detected.

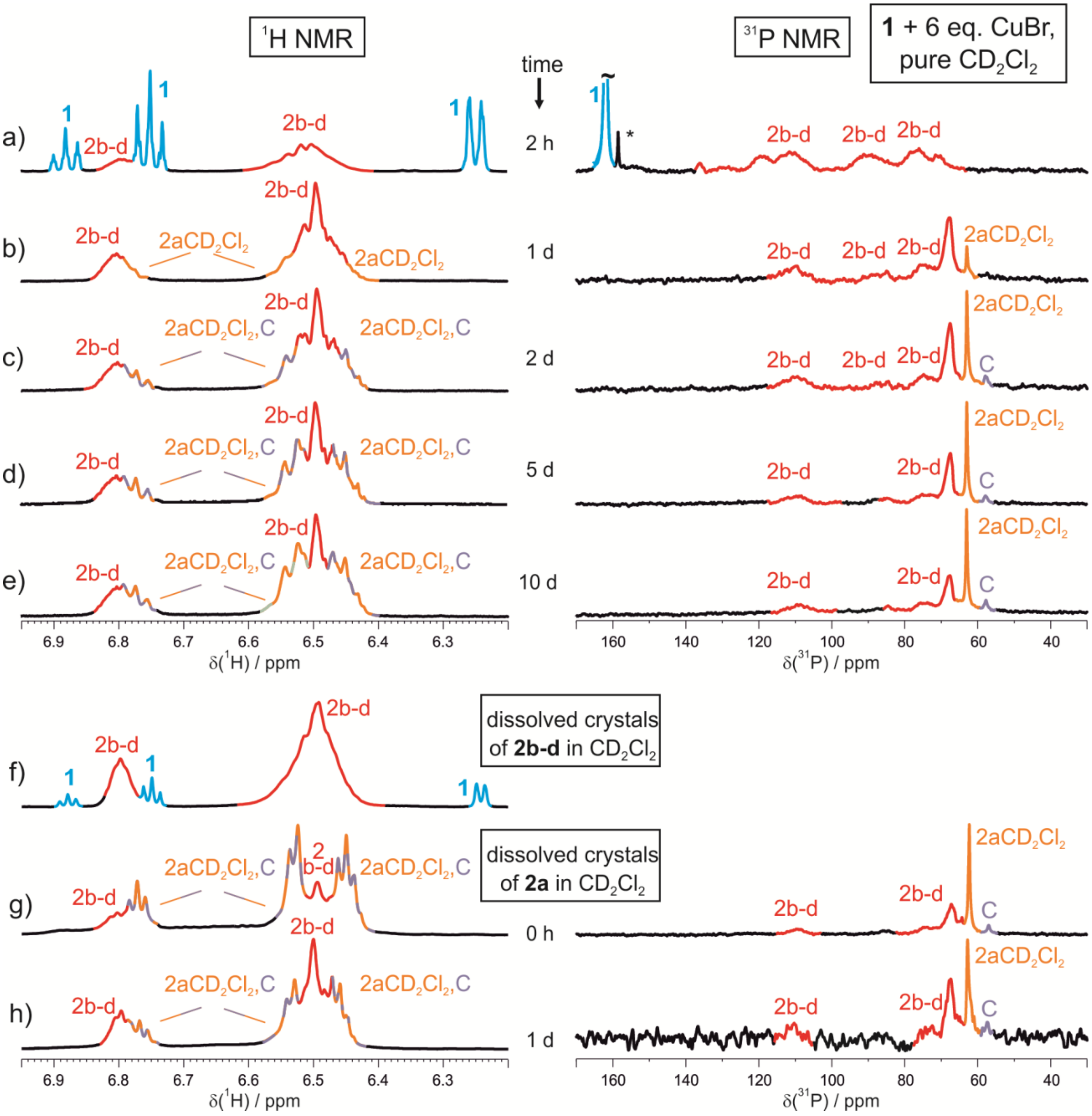

Fig. S 7. ^{1}H (left) and ^{31}P spectra (right) of samples containing **1** and six equivalents of CuBr in pure CD$_2$Cl$_2$ after varying reaction times at 300 K and 400 MHz; dissolved crystals of f) **2b-d** and g) **2a** and h) **2a** after 1 d in solution, each at 300 K and 600 MHz in pure CD$_2$Cl$_2$.

These results and assignments are corroborated by solving **2b-d** and **2a** crystals in CD_2Cl_2. **2b-d** crystals yielded **2b-d** and **1** (see Fig. S 7f). Directly after dissolving **2a** crystals in CD_2Cl_2 (see Fig. S 7g), not only **2aCD₂Cl₂** but also considerable amounts of **2b-d** and small amounts of C are formed in solution, indicating that from some of the **2a** crystals CuBr is released to form **2b-d**. After one day in solution (see Fig. S 7h), the inverse trend to the formation process in pure CD_2Cl_2 (see Fig. S 8a-e) is detected. **2b-d** increases at the expense of **2a**$\mathbf{CD_2Cl_2}$, C remains constant. These experiments demonstrated, that due to the low solubility of CuBr in pure CD_2Cl_2 and the equilibria, which exist in solution, pure **2b-d** and **2a** crystals are not stable but that CuBr units are removed from the supramolecules and additionally **1** and **2b-d** are formed respectively.

3.3 Acetonitrile titrations to 2a and 3 crystals dissolved in CD_2Cl_2

The addition of increasing amounts of CD_3CN to **2a** and **3** crystals dissolved in CD_2Cl_2 confirms the assignments of the supramolecules, the existence of both **2a**$\mathbf{CD_2Cl_2}$ and **2a**$\mathbf{CD_3CN}$ beside each other in solution, the reversible formation of the various supramolecules depending on the amount of CuBr and CD_3CN present and the preferred formation of **2a**$\mathbf{CD_3CN}$ even in the presence of only small amounts of CD_3CN (see Fig. S 8 and S 8). The spectrum of **2a** crystals dissolved in CD_2Cl_2 show not only the sharp signals of **2a**$\mathbf{CD_2Cl_2}$ but also considerable amounts of **2b-d** and small amounts of C as discussed above (see Fig. S 7g and S 7a). Upon addition of 4 vol% CD_3CN (see S 7b), the relative amount of **2b-d** decreased slightly and small amounts of **1** appeared. However, the most prominent change is the appearance of the broad ^{31}P-signal of **2a**$\mathbf{CD_3CN}$ in the ^{31}P spectrum and the sharp and structured ^{1}H signals of **2a**$\mathbf{CD_3CN}$ in the proton spectrum both with large integrals, while the ^{1}H and ^{31}P signals of **2a**$\mathbf{CD_2Cl_2}$ are still present. This indicates that **2a**$\mathbf{CD_3CN}$ is preferentially formed even at low amounts of CD_3CN in solution (the lower energy of **2a**$\mathbf{CD_3CN}$ is also in agreement with the preferred crystallization of **2a** from mixtures of CH_2Cl_2 and CH_3CN). At 9 vol% CD_3CN the ^{1}H and ^{31}P signals of **2a**$\mathbf{CD_2Cl_2}$ are nearly vanished and **2a**$\mathbf{CD_3CN}$ is the dominant species (see Fig. S 8c). At higher CD_3CN concentrations the amount of **2b-d** and **1** slightly increased, indicating a decomposition of the supramolecules at higher amounts of acetonitrile.

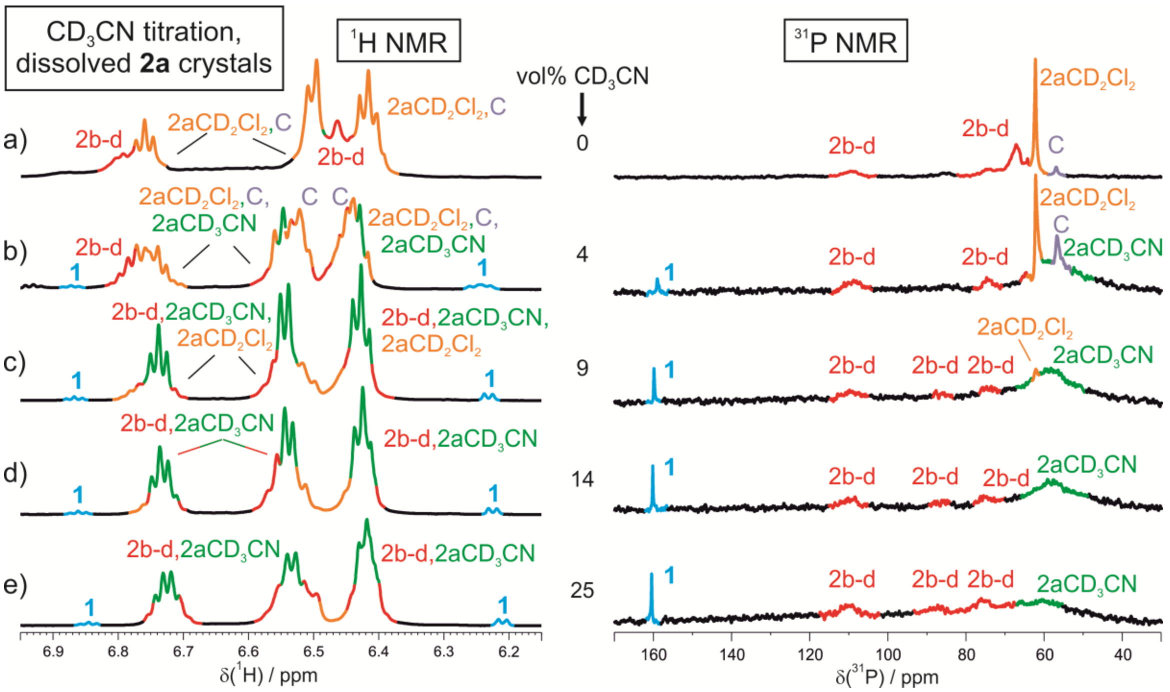

Fig. S 8. ^{1}H (left) and ^{31}P spectra (right) of dissolved **2a** crystals in CD_2Cl_2 with increasing vol% of CD_3CN at 298 K and 600 MHz.

These trends were corroborated by the ^{1}H spectra obtained from a CD_3CN titration starting with crystals of **3** dissolved in pure CD_2Cl_2 (see Fig. S 9). **3** itself was not detected in considerable amounts because the concentration of CD_3CN necessary for the formation of **3** was too low. However, due to the high amount of CuBr in **3** now strong signals of **2aCD₂Cl₂** and only small signals of **2b-d** were detected. With 4 vol% CD_3CN, 44 % of **3**, 29 % of **2aCD₃CN** and 27 % of **2aCD₂Cl₂** were observed indicating the re-assembling of **3** and, the strong preference of **2aCD₃CN** over **2aCD₂Cl₂**, and the loss of CuBr upon dissolution. At higher contents of CD_3CN much CuBr units are distached, thus **3** and **2aCD₂Cl₂** were reduced and **2aCD₃CN** appeared as main species beside small amounts of **1**.

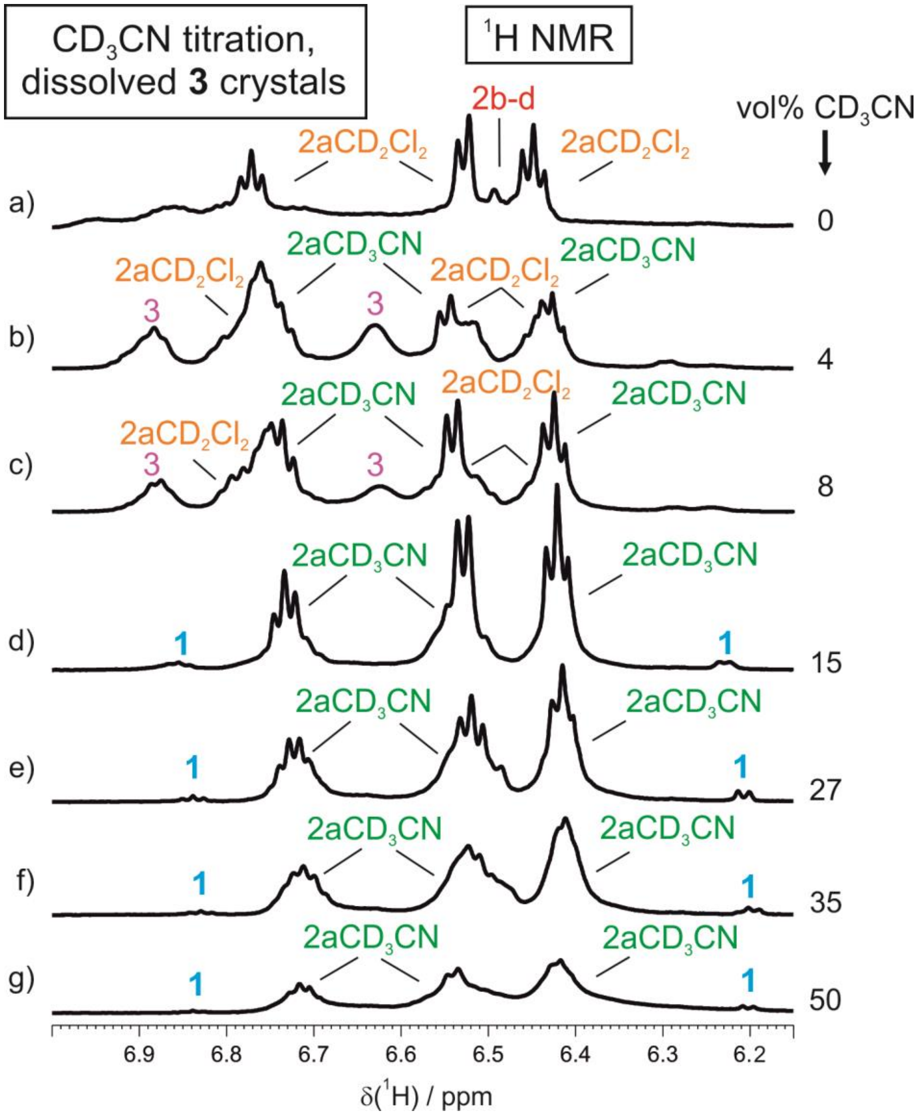

Fig. S 9. ^{1}H spectra of dissolved **3** crystals in CD_2Cl_2 with increasing vol% of CD_3CN: a) 0 vol%, b) 4 vol%, c) 8 vol%, d) 15 vol%, e) 27 vol%, f) 35 vol%, g) 50 vol%, each at 298 K and 600 MHz.

Furthermore, the influence of CD_3CN was also investigated in a CuBr titration similar to the one depicted in Fig. S 5 but with 14 instead of 9 vol% CD_3CN in CD_2Cl_2 (see Fig. S 10). There, the CD_3CN concentration is too high to observe **2aCD₂Cl₂**. Again the CuBr dependent stepwise formation of **2b-d**, **2aCD₃CN** and **3** and the optimal conditions for the formation of **3** are visible.

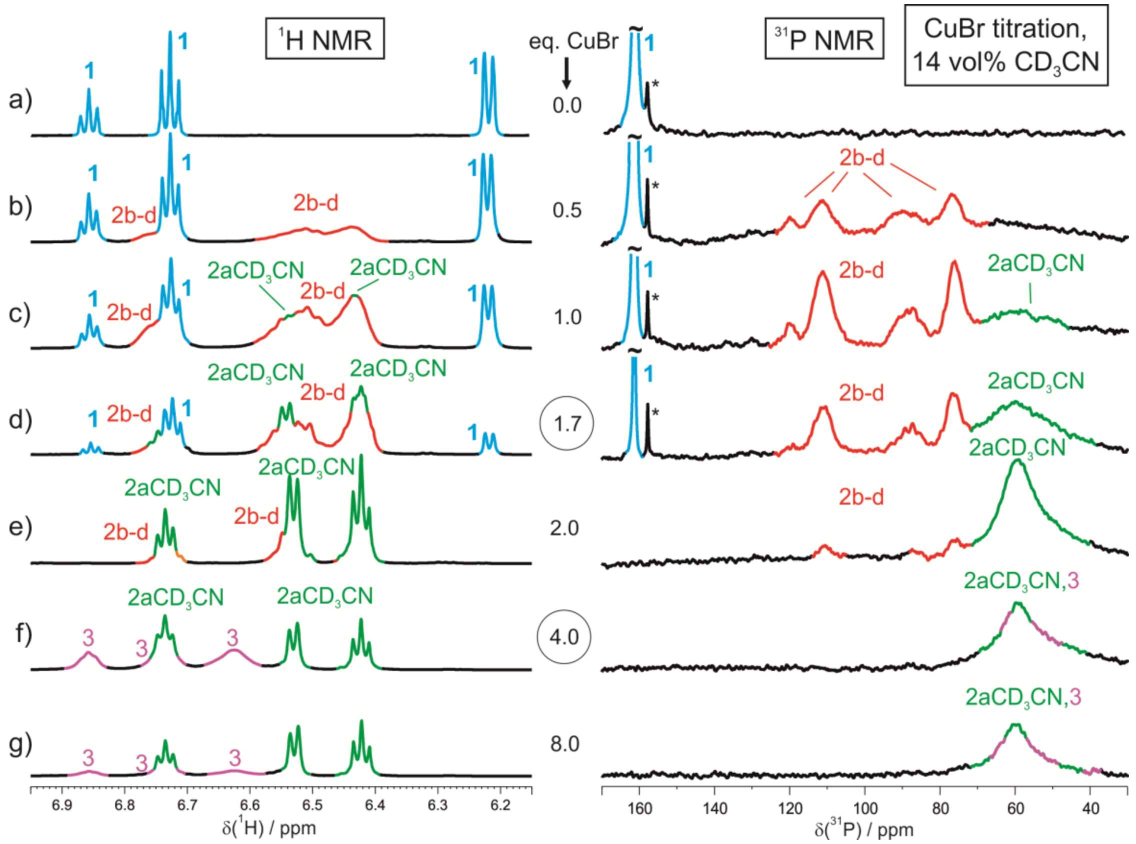

Fig. S 10. ^{1}H (left) and ^{31}P spectra (right) of samples containing **1** and increasing equivalents of CuBr in CD_2Cl_2 with 14 vol% CD_3CN at 298 K and 600 MHz.

3.4 Size estimation from DOSY and X-ray data

From diffusion-ordered spectroscopy (DOSY) experiments (see Fig. S 11)[5-8] the translational self-diffusion coefficients D of molecules in solution can be calculated according to the Stejskal-Tanner equation.[4,5,8,9] With the diffusion coefficients D of the analyte and of TMS (acting as viscosity reference), the hydrodynamic radius r_H (and there of the hydrodynamic volume V_H) of the analyte can be estimated following the Stokes-Einstein equation.[10]

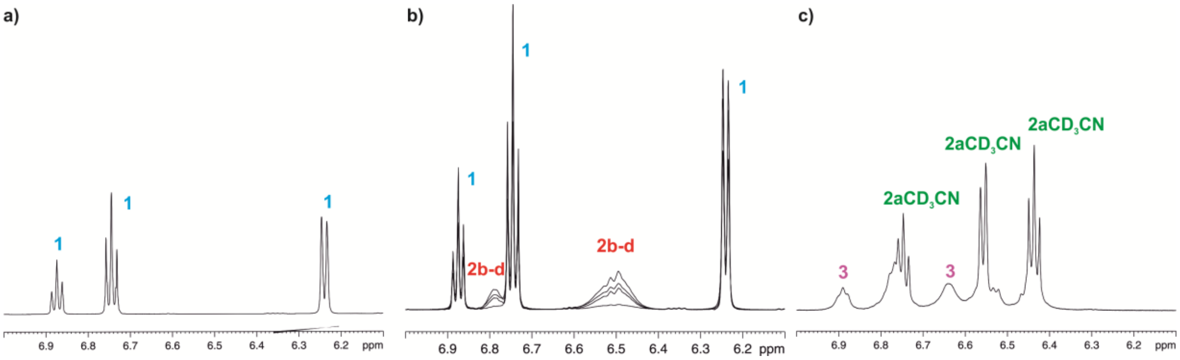

Fig. S 11. ^{1}H NMR spectra used of the different species at 298/300 K and 600 MHz (**1**, **2b-d** in CD_2Cl_2; **2aCD$_3$CN**, **3** in CD_2Cl_2/CD_3CN) for ^{1}H-DOSY studies.

Table S 7. Diffusion coefficients and hydrodynamic volumes of the different species.

Species	D / 10^{-10} m^2 s^{-1}	V_H / 10^3 Å^3
**1**	10.69 ± 0.13	0.7 ± 0.1
**2b-d**[a]	3.98 ± 0.05	10.7 ± 0.4
**2aCD$_3$CN**	3.81 ± 0.02	12.1 ± 0.2
**3**	3.49 ± 0.03	15.6 ± 0.4

[a] average of all subspecies.

2b-d provides in the ^{31}P-spectra several separated signals but only overlapping signals in the ^{1}H spectra. Reliable DOSY spectra of slow diffusing supramolecules on ^{31}P-signals with such broad linewidths are not possible. Therefore, only ^{1}H-DOSY studies on the overlapped proton signal representing all subspecies of **2b-d** were done. The resulting ^{1}H-DOSY-derived hydrodynamic volume of **2b-d** of 10.7±0.4·10³ Å³, averaged over all subspecies, was 15 times larger than that of **1** (0.7·10³ Å³) and 5.5·10³ Å³ smaller than the crystal-derived volume of $[\{Cp^{Bn}Fe(\eta^5\text{-}P_5)\}_{12}\{CuBr\}_{20-n}]$ or $[\{Cp^{Bn}Fe(\eta^5\text{-}P_5)\}_{12}\{CuBr\}_{20}]$, V_{cryst}(**2**) = 16.2·10³ Å³ (see Table S 7).

In the corresponding NOESY spectrum, the signals of **1** were in the extreme narrowing limit (negative sign of the cross peaks) indicating a fast reorientation typical for small molecules. In accordance with the DOSY results, the signals of **2b-d** were in the slow tumbling limit (positive cross peaks) indicating a large hydrodynamic radius. Furthermore, exchange peaks between **1** and **2b-d** were detected in the NOESY/ROESY spectra of this sample.

The DOSY data, the sum of the NMR spectra and the crystallographic data assemble now to a picture of the formation and the relative stabilities of these supramolecules. In the ^{31}P spectra always a distinct set of signals were detected for **2b-d** with some intensity variations depending on the actual CuBr concentration. The DOSY data of the averaged ^{1}H signal of several **2b-d** species show a large hydrodynamic volume 15 times larger than that of **1** (even in the presence of chemical exchange with **1** leading usually to apparently faster diffusion) and in the crystals all the time the same scaffold with different occupation of the CuBr sites is observed. These data suggest a preferred formation and a high stability of the **2b-d** scaffold in solution and speak clearly against a continuous stepwise formation of this supramolecular scaffold.

The relative hydrodynamic volumes of **1**, **2** and **3** correlate to those calculated from crystallographic data but the absolute values deviate. Thus, from crystallographic data the volume of the monomer was estimated to be V_{cryst}(**1**)=589 Å³. In addition, the diameter of the **2** cluster was estimated to be d = 31.4 Å from the maximum H-H distance plus twice the Van-der-Waals radius of a proton (1.2 Å) *(24)*. Assuming a perfect spherical geometry the volume is calculated to be V_{cryst}(**2**)=16.2·10³ Å³. For the **3** cluster, from crystallographic data a diameter of d = 35.6 Å can be estimated and the also globular volume can therefore be valued to V_{cryst}(**3**)=23.6·10³ Å³. These deviations may be caused by the way of calculation, because other calculations of the hydrodynamic volume from crystallographic data led to V_{cryst}(**1**) = 0.6·10³ Å³, V_{cryst}(**2**) = 13.2·10³ Å³ and V_{cryst}(**3**) = 17.5·10³ Å³ in accordance with the NMR data.

3.5 Encapsulation of guest molecules

First the encapsulation of ferrocene was tested in a solvent mixtures providing **2aCD_3CN** and **3** (**1** and 2 eq. CuBr in CD_2Cl_2 with about 3 vol% CD_3CN) and indeed, a small signal at 0.66 ppm was detected indicating the encapsulation of ferrocene in **2aCD_3CN** (see Fig. S 12b).

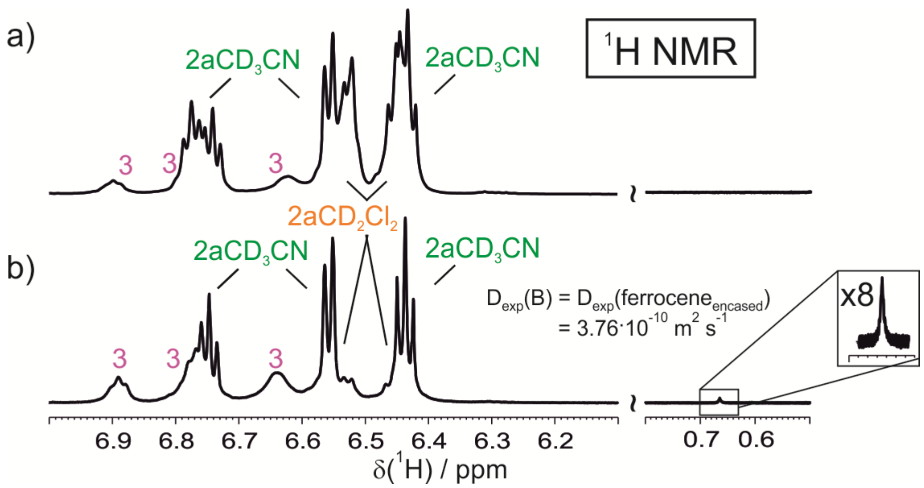

Fig. S 12. ^{1}H spectra of **1** + two eq. CuBr in CD_2Cl_2/CD_3CN samples a) without and b) with ferrocene.

Due to the strong shielding by the globular host, the incorporation of a guest should be indicated by an upfield shift of the signal of an encased molecule compared to the free compound, which was observed for the encapsulation of *o*-carborane (5 ppm in the $^{11}B\{^{1}H\}$-MAS spectrum)[11] and of C_{60} fullerene (3 ppm in the ^{13}C spectrum).[12] In addition, host and guest must have the same diffusion coefficient and proton containing guests should show NOESY cross peaks to the host. In principle, TMS (added as internal viscosity standard), CD_3CN, CD_2Cl_2 or ferrocene could be incorporated in the container molecules, but incorporated solvent or TMS molecules were not detected in previous control experiments, although an encapsulation of CD_2Cl_2 inside **2** crystals was supposed from X-ray analysis. This might be attributed to the expected low signal intensities of such incorporated solvents, since perdeuterated solvents with a high degree of deuteration (99.8 % or higher) were used. Furthermore, the ^{1}H signal intensities of the non-perdeuterated fraction of CHD_2CN and $CHDCl_2$ are distributed over their multiplet fine structure.

No NOESY cross peaks from 0.66 ppm to the signals **2aCD$_2$Cl$_2$** or **3** were observed.

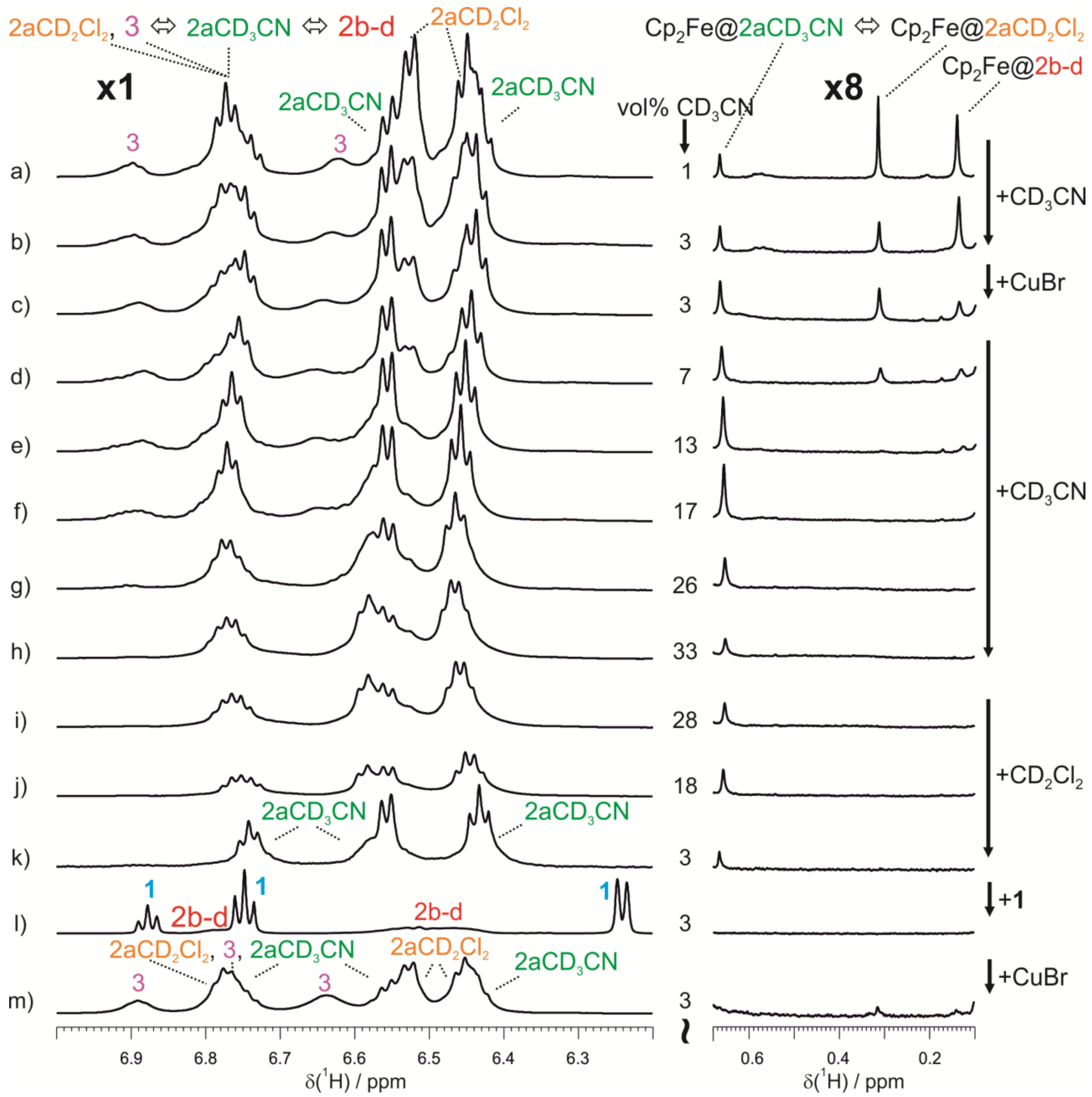

Fig. S 13. ^{1}H spectra of the self-assembling supramolecular switch with incorporated guests composed of **1**, 2.0 eq. CuBr and 0.25 eq. ferrocene in CD_3CN/CD_2Cl_2 mixtures, each at 298 K and 600 MHz. The intensities of the ^{1}H sections on the right side are increased by a factor of eight. Please be aware that due to the dilution procedure the relative intensities of the guests have to be corrected by the absolute intensities of the guest molecules. Therefore, a direct "vertical" comparison is misleading (e.g. the percentage of encapsulated guests is similar in j) and f)).

Together with the encapsulation also a significant shift from **2aCD$_2$Cl$_2$** to **2aCD$_3$CN** was observed (see Fig. S 13). However, from these experiments, it was not clear whether this might be attributed to small variations of the amount of CD_3CN in the sample with ferrocene or whether **2aCD$_2$Cl$_2$** is not able to encapsulate ferrocene. Therefore, a titration row with increasing amounts of CD_3CN and ferrocene was measured (see Fig. S 13; **1**, 2.0 eq. CuBr, 0.25 eq. ferrocene in CD_2Cl_2 with ca. 1 vol% CD_3CN stirred for several hours). In this setup, a potential crystallization of one of the supramolecules and, thus, potential shift of the thermodynamic equilibrium was avoided by the use of relatively low concentrations and could be finally excluded by the use of an external NMR standard. Therefore, all changes in the relative amounts of the supramolecules are clearly attributable to a transformation of the clusters into one another. With 1 vol% CD_3CN as expected a very high amount of **2aCD$_2$Cl$_2$** was detected beside **2aCD$_3$CN** and **3**. In addition, three small singlets at 0.66, 0.31 and 0.13 ppm were detected (see Fig. S12a; outside this chemical shift range very intense signals are present in the ^{1}H spectrum originating from TMS and grease-like compounds, thus further encapsulation signals cannot be excluded). All three signals show chemical shift values and diffusion coefficients indicating the encapsulation of ferrocene into the supramolecular scaffold of **2** (diffusion coefficients for the signals at 0.66, 0.31 and 0.13 ppm are 3.61, 3.80 and 3.73 $*10^{-10}m^2/s$, while those of **2aCD$_3$CN**, **2aCD$_2$Cl$_2$** and **3** are 3.60, 3.87 and 3.45 $*10^{-10}m^2/s$). Upon further addition of CD_3CN **2aCD$_3$CN** increased at the expense of **2aCD$_2$Cl$_2$** and accordingly the singlet at 0.66 ppm increased while the signals at 0.31 and 0.13 ppm vanished. This corroborated the assignment of the signal at 0.66 ppm as Cp_2Fe@**2aCD$_3$CN**. In addition a NOESY spectrum was measured at 18 vol% of CD_3CN, which provided a relatively high amount of Cp_2Fe@**2aCD$_3$CN** and allowed the detection of an NOE cross peak from the methylene protons of **2aCD$_3$CN** to the signal at 0.66 ppm (see Fig. S 14). Neither cross peaks from 0.66 ppm to the aromatic signals of **2aCD$_3$CN** nor to **3** were observed (higher amounts of **3** in this sample see Fig. S13) confirming the assignment of the signal at 0.66 ppm to Cp_2Fe@**2aCD$_3$CN**.

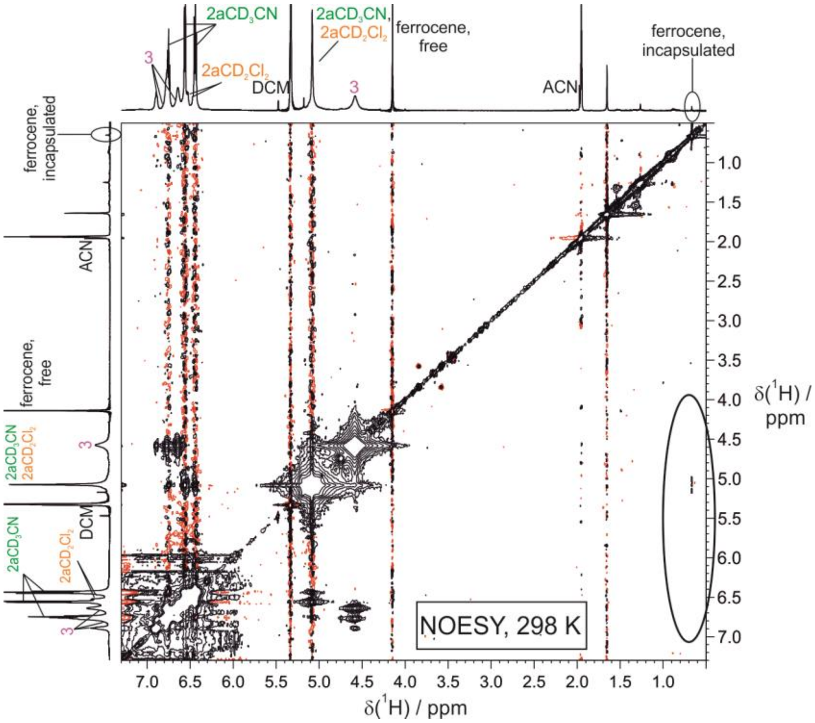

Fig. S 14. NOESY spectrum of **1**+six eq. CuBr+ferrocene in CD_2Cl_2 with 18 vol% CD_3CN at 298 K and 600 MHz. A cross peak from an encapsulated ferrocene guest to the methylene protons of **2aCD$_3$CN** could be detected.

Upon addition of up to 17 vol% CD_3CN the signals at 0.31 and 0.13 ppm vanished together with **2aCD$_2$Cl$_2$** identifying these signals as Cp_2Fe@**2CH$_2$Cl$_2$** (see Fig. S 13). From 1 to 3 vol% of CD_3CN the signal at 0.31 decreased whereas that at 0.13 ppm remained. Considering the formation trends of these supramolecules from **2aCD$_2$Cl$_2$** to **2b-d** due to the dissolution of CuBr units upon addition of CD_3CN, this suggested strongly the assignment of the signal at 0.31 ppm to Cp_2Fe@**2aCH$_2$Cl$_2$** and of 0.13 ppm to Cp_2Fe@**2b-dCH$_2$Cl$_2$**. To test this, CuBr was added (see Fig. S 12b and c). Indeed, as expected the signal of Cp_2Fe@**2b-dCH$_2$Cl$_2$** at 0.13 ppm decreased strongly, while that of Cp_2Fe@**2aCH$_2$Cl$_2$** at 0.31 ppm increased together with that of Cp_2Fe@**2aCD$_3$CN** at 0.66 ppm most probably due to a slightly higher amount of CD_3CN.

Next the reversibility of the encapsulation was tested by reducing again the amount of CD_3CN as well as adding again **1** and CuBr to compensate the dilution effect (see Fig. S 13). The signals of the encapsulated ferrocene Cp_2Fe@**2aCD$_3$CN** at 0.66 ppm, Cp_2Fe@**2aCH$_2$Cl$_2$** at 0.31 and Cp_2Fe@**2b-dCH$_2$Cl$_2$** at 0.13 ppm follow exactly the formation trends of the respective supramolecules (for Cp_2Fe@**2b-dCH$_2$Cl$_2$** a species close to Cp_2Fe@**2aCH$_2$Cl$_2$** seems to be

required because spectra S 12 and S 14 did not show any encapsulation signals of these species) and the spectra show a complete reversibility in this cyclus. Note, that a release of ferrocene is also enabled by the switch of **Cp_2Fe@2-Br** to **3**. To do so, crystals of **Cp_2Fe@2-Br** are dissolved in CH_2Cl_2 and 9 vol% CH_3CN and an excess of CuBr is added. Layering with toluene leads to a quantitative crystallization of the supramolecule **3**, which has no accessible void for the guest molecule, while ferrocene remains in solution.

References

1. F. Dielmann, R. Merkle, S. Heinl, M. Scheer, *Z. Naturforsch. B* **2009**, *64*, 3-10.
2. V. A. Blatov, *Newslett. Comm. Cryst. Comp. IUCr.* **2006**, 4-38. (available at http://www.iucr.org/iucr-top/comm/ccom/newsletters/).
3. R. Kleinmaier, P. Igel, M. Keller, A. Buschauer, R. M. Gschwind, *J. Am. Chem. Soc.* **2010**, *132*, 11223-11233.
4. A. Jerschow, N. Muller, *J. Magn. Reson.* **1997**, *125*, 372-375.
5. C. S. Johnson, Jr., *Prog. Nucl. Magn. Reson. Spectrosc.* **1999**, *34*, 203-256.
6. Y. Cohen, L. Avram, L. Frish, *Angew. Chem., Int. Ed.* **2005**, *44*, 520-554.
7. W. S. Price, *Concepts Magn. Reson.* **1997**, *9*, 299-336.
8. W. S. Price, *Concepts Magn. Reson.* **1998**, *10*, 197-237.
9. E. O. Stejskal, J. E. Tanner, *J. Chem. Phys.* **1965**, *42*, 288-292.
10. A. Macchioni, G. Ciancaleoni, C. Zuccaccia, D. Zuccaccia, *Chem. Soc. Rev.* **2008**, *37*, 479-489.
11. M. Scheer, A. Schindler, C. Gröger, A. V. Virovets, E. V. Peresypkina, *Angew. Chem., Int. Ed.* **2009**, *48*, 5046-5049.
12. M. Scheer, A. Schindler, R. Merkle, B. P. Johnson, M. Linseis, R. Winter, C. E. Anson, A. V. Virovets, *J. Am. Chem. Soc.* **2007**, *129*, 13386-13387.
